# Survival at the edge: genomic vulnerability and genetic purging of a limestone cliff-endemic sky island shrub under climate change

**DOI:** 10.48130/forres-0026-0010

**Published:** 2026-04-14

**Authors:** Liao-Cheng Zhao, Wei-Long Gao, Nan Wang, Han-Ze Gu, Yuan-Mi Wu, Zi-Xiang Xu, Zhi-Hong Ma, Xian-Yun Mu

**Affiliations:** 1 College of Ecology and Nature Conservation, Beijing Forestry University, Beijing 100083, China; 2 Administration of Beijing Miyun Wuling Mountain Nature Reserve, Beijing 101506, China

**Keywords:** Climate change, Demographic history, Genetic load, Genomic offset, Local adaptation, *Lonicera oblata*

## Abstract

Climate change poses a significant threat to biodiversity, highlighting the urgent need to understand species' adaptive potential. Using the sky island limestone-endemic shrub *Lonicera oblata* in North China as a model, we integrated genomic, transcriptomic, and metabolomic analyses to investigate its evolutionary trajectory. The assembled genome is 786.92 Mb in size, and it has the highest proportion of repetitive sequences (66.47%) in *Lonicera*. Multiple expanded gene families were enriched in pathways related to stress response, including oxidoreductase activity, cell wall synthesis, and energy metabolism. The bHLH gene family exhibits both a significant expansion in the comparative genomic analysis and a convergent transcriptional activation under calcium stress, correlating with the metabolic reprogramming of organic acid synthesis and ion homeostasis. We detected low genetic diversity (*π*: 2.24e–3 to 2.80e–3), high differentiation (average fixation index: 0.16), drastic historical decline, and strong genetic load among populations. Notably, the northeasternmost and most recently diverged population (Jiankou) exhibited extreme inbreeding but the lowest genetic load, suggesting that genetic purging enhances small population survival. The genotype-environment association analysis identified 1,286 core SNPs potentially correlated with local adaptation. Genomic offset projections predicted high maladaptation risk under future climates, especially in eastern and southern populations. This study provides essential insights into the mechanisms of local adaptation, genomic vulnerability, and climate resilience of threatened sky island species, and offers guidance for targeted conservation strategies.

## Introduction

Global warming threatens Earth's biodiversity, accelerating species extinctions, distributional shifts, and severe disruptions to ecosystem functions^[1,2]^. Species typically respond through dispersal to more suitable habitats or local adaptation via phenotypic plasticity and genetic variation^[3,4]^. This adaptation relies on sufficient genetic variation and population connectivity. However, the response capacity of locally endemic species with small populations remains largely unknown.

Mountains harbor a disproportionate global biodiversity, yet the underlying mechanisms remain unclear^[5−7]^. Sky islands are geographically isolated peaks surrounded by lowland barriers, which restrict species migration due to unfavorable environmental conditions^[8]^. Species with such distribution patterns are observed in the Rocky Mountains, the Alps, and the Himalaya-Hengduan Mountains^[9]^, which are pivotal for studying montane biodiversity evolution^[10,11]^. Their populations often exhibit habitat fragmentation, low genetic diversity, and high genetic differentiation. Historical climate oscillations and anthropogenic pressures further disrupt the evolutionary trajectories of sky island species^[8,12]^. Despite this, empirical evidence linking climate change, habitat specialization, and genetic adaptation remains scarce, particularly for sky island species endemic to montane regions.

As a critical but understudied refugial region, the North China Mountains (NCM) face multiple threats, especially against the backdrop of climate change and anthropogenic pressure. Comprising the north-northeast-trending Taihang Mountains and east-west-trending Yanshan Mountains, the NCM bridges northern China's second- and third-tier terrain and serves as a biological corridor linking Northeast and South China. NCM features complex geological structures with deep canyons, vertical cliffs, and pronounced microenvironmental heterogeneity characterized by abundant limestone^[13]^. Locally endemic limestone species in this area confront severe threats from habitat fragmentation, population differentiation, and climate change, such as *Taihangia rupestris* T. T. Yu & C. L. Li, and *Opisthopappus taihangensis* (Y. Ling) C. Shih^[14,15]^. Information on both the historical accumulation of disadvantageous genetic variants (genetic load) and the risk of maladaptation to future global warming (genomic offset) is vital for endangered species conservation^[16,17]^. However, they are poorly investigated for limestone-endemic species in the NCM, hindering effective conservation.

The NCM-endemic limestone-dwelling shrub, *Lonicera oblata* K. S. Hao ex P. S. Hsu & H. J. Wang, persists in seven fragmented populations located atop mountain ridges^[18]^. Species distribution modeling indicated climate sensitivity with northward centroid migration that favored low-temperate conditions since the Last Interglacial^[19]^. Restriction site-associated DNA sequencing revealed low genetic diversity but high differentiation among and within populations^[20]^, partly attributed to fragmented populations, limited gene flow, and mixed outbreeding/self-fertilization^[21]^. Geographic barriers, including the Loess Plateau (west), the Yinshan Mountains (north), and the Bohai Sea (east), constrain its expansion, driving an extinction vortex. It was listed as one of the national key protected wild plants of China in 2021^[22]^. This combination of a sky island distribution pattern, complex environmental gradients, fragmented habitat, and strong genetic divergence among populations makes *L. oblata* an ideal system for elucidating how limestone-endemics adapted and persisted in mountainous landscapes.

In this study, we employed multiple genomic analyses leveraging a chromosome-level *de novo* assembly of *L. oblata* and whole-genome resequencing of 140 individuals to: (1) characterize genome composition and population genetic patterns; (2) elucidate the mechanism of local adaptation to the limestone habitat; and (3) assess adaptive potential under future climate change to inform species conservation. Our findings provide insights into the evolution of limestone-adapted genomes and advance conservation efforts for endangered NCM cliff species.

## Materials and methods

### Genome sequencing, assembly, and annotation

We collected fresh leaves of *L. oblata* from the Jiankou (JK) population (Huairou District, Beijing, China) for *de novo* genome sequencing with official authorization. After extracting total genomic DNA using the CTAB protocol^[23]^, we verified its integrity using 0.8% agarose gel electrophoresis. Sequencing was performed using Illumina NovaSeq 6000 (150-bp paired-end), PacBio Revio (HiFi reads), and Hi-C libraries (Illumina NovaSeq 6000). Genome size and heterozygosity were estimated using *k*-mer analysis. To ensure comprehensive genome coverage, we processed the raw sequencing data by performing adapter trimming and quality filtering with Fastp v0.23.4^[24]^. Hifiasm v0.16.1^[25]^ generated the primary assembly using default OLC parameters, followed by chromosome-level scaffolding with Hi-C data. We assessed genome quality via BUSCO v5.3.0^[26]^ and BWA-MEM v0.7.17^[27]^, respectively.

We utilized multi-organ transcriptomic sequencing data from roots, stems, flowers, and fruits to assist in genome annotation. We performed genome-wide repetitive annotation and genome structural annotation through integrated analyses of both homology-based approaches and *de novo* prediction methods^[28,29]^. We estimated the insertion time of long terminal repeats (LTRs) using EDTA (https://github.com/oushujun/EDTA.git). For functional annotation, we conducted BLASTP alignments (E-value < 1.0e–5) against the Gene Ontology (GO) database^[30,31]^ and the Kyoto Encyclopedia of Genes and Genomes (KEGG) database^[32]^.

### Comparative genomic analyses

We selected 14 species for comparative genomic analyses, including five species from the Caprifoliaceae, i.e., *L. caerulea* L., *L. japonica* Thunb., *L. maackii* (Rupr.) Maxim., *L. macranthoides* Hand.-Mazz., and *Heptacodium miconioides* Rehder. Specifically, we included three limestone-endemic species distributed in karst habitats in Southwest China (*Platycarya longipes* Y. C. Wu, *Marsdenia tenacissima* [Roxb.] Moon), and central China (*Corydalis tomentella* Franch.). *Amborella trichopoda* Baill. was selected as the outgroup. We obtained their genomic data from NCBI (www.ncbi.nlm.nih.gov) and NGDC (https://ngdc.cncb.ac.cn/gwh).

We performed orthogroup clustering and identified single-copy orthologs using OrthoFinder v2.2^[33]^. After extracting conserved sequences from the aligned single-copy genes by trimming and filtering, we reconstructed a maximum likelihood-based phylogenetic tree using IQ-TREE v2.4.0^[34]^. We analyzed the expansion and contraction patterns of orthogroups using CAFE 5^[35]^. Additionally, we inferred a whole-genome duplication (WGD) event by calculating the ratio of nonsynonymous (*Ka*) to synonymous (*Ks*) nucleotide substitution rates. The *Ka* and *Ks* values were calculated using WGDI^[36]^. The mutation rate (μ) was calculated as μ = *Ks*/2T^[37]^, yielding a rate of 1.43e–9. This was based on a *Ks* value of 0.06169 between *L. oblata* and *L. caerulea*, and the divergence time (T) between these two species. We determined genome syntenic relationships and identified homologous chromosomes using MCscanX^[38]^, and compared genome structures with the software SyRI v1.4^[39]^. We predicted transcription factor families via PlantTFDB (https://planttfdb.gao-lab.org/) and further identified putative families through BLAST alignment and conserved domain screening. Subsequently, we constructed a phylogenetic tree and performed chromosomal localization using TBtools v2.31^[40]^.

### Transcriptomic-metabolomic association analyses

We performed transcriptomic-metabolomic association analyses in response to calcium stress treatment. We applied 500 mL of 300 mmol·L^−1^ CaCl_2_ solution to healthy five-year-old seedlings to induce stress. We collected leaf samples at 0 (CK), 1, 3, 6, 12, and 24 h, immediately froze them in liquid nitrogen, and stored the samples at −80 °C. We extracted total RNA from the leaves using TRIzol reagent (Invitrogen, USA), and constructed cDNA libraries with the TruSeq™ RNA Sample Preparation Kit (Illumina, USA). We sequenced these libraries on an Illumina NovaSeq 6000 platform (PE150), analyzing three biological replicates per time point. We processed the raw reads with Fastp v0.23.4^[24]^, aligned them to the reference genome using HISAT2^[41]^, and quantified gene expression with featureCounts^[42]^. The gene expression was normalized as fragments per kilobase of transcript per million mapped reads (FPKM). We identified differentially expressed genes (DEGs) based on |log_2_(Fold Change)| ≥ 1 and a false discovery rate (FDR) < 0.05.

We adopted the untargeted metabolomics method of Liu et al.^[43]^. Leaf extracts (in cold methanol : acetonitrile : water, 2:2:1) were separated on a HILIC column using UHPLC-MS/MS, with both positive and negative ionization. Intermittent quality control (QC) runs were performed using a sample pooled from all extracts. We converted the raw data using ProteoWizard^[44]^, then processed it with XCMS^[45]^ for peak picking and alignment. We matched the features against an in-house database (Shanghai Applied Protein Technology Co., Ltd, Shanghai, China)^[46,47]^, applying a mass error tolerance of < 10 ppm. We removed features with > 50% missing values, imputed the remaining missing values using a KNN method, and filtered out features with an RSD > 50% in the QC samples. We selected differentially accumulated metabolites (DAMs) based on multivariate analysis, requiring VIP > 1.0, *p* < 0.05, and |FC| > 1.0. In the association analysis, we annotated and performed KEGG enrichment analysis on the DEGs and DAMs to identify co-enriched pathways.

### Whole-genome resequencing and population genetic analyses

We collected 140 individuals from seven natural populations across the entire distribution range of *L. oblata*, i.e., Heduling (HDL), Wutaishan (WTS), Bijiashan (BJS), Jimingshan (JMS), Baihuashan (BHS), Songshan (SS), and JK. Whole-genome resequencing was performed on the Illumina NovaSeq 6000 platform. For data processing, we initially trimmed the raw sequences with Fastp v0.23.4 to remove adapters and low-quality reads, thereby obtaining high-quality reads. We then mapped these reads to our *de novo-*assembled *L. oblata* reference genome using the BWA-MEM algorithm of BWA v0.7.17 with default parameters. After sorting the alignment files using SAMtools v1.9^[48]^, we marked and removed duplicate reads generated during sequencing with Picard v2.18 (http://broadinstitute.github.io/picard/). We identified single-nucleotide polymorphisms (SNPs) using GATK v4.0.5.1^[49]^ and its modules (HaplotypeCaller, CombineGVCFs, and GenotypeGVCFs) to generate a merged VCF file, which we further refined using VCFtools v1.9^[50]^ with the parameters '--min-meanDP 3 --minGQ 10 --minQ 30 --maf 0.05 --remove indels --max-missing 0.8 --min-alleles 2 --max-alleles 2'. Finally, we obtained a high-quality SNPs dataset for downstream analyses.

To minimize the effect of linkage disequilibrium (LD) in population structure analyses, we performed genome-wide LD pruning using PLINK v1.9^[51]^ with the parameters '--indep-pairwise 100 1 0.5', yielding a set of LD-pruned SNPs for three analyses: (1) population structure analysis with Admixture v1.3.0^[52]^, which evaluated cross-validation error for *K* = 1–10; (2) principal component analysis (PCA) implemented in PLINK v1.9 with default parameters, and (3) phylogenetic reconstruction, which involved converting from vcf to FASTA format using vcf2phylip.py (https://github.com/edgardomortiz/vcf2phylip). We then built a maximum likelihood tree and calculated clade support values with 1,000 replicates via IQ-TREE^[34,53]^, selecting *L. webbiana* Wall. ex DC. as the outgroup. We calculated the population fixation index (*F*_ST_) using VCFtools v1.9 in 100-kb sliding windows with 10-kb steps, while nucleotide diversity (*π*) and Tajima's *D-*value were calculated in corresponding 100-kb windows. We identified private alleles among the seven populations based on the LD-pruned SNPs dataset using VCFtools v1.9. We estimated the LD decay pattern by calculating the squared correlation coefficient (*r*^2^) between pairwise SNPs within 300-kb windows using PopLDdecay v3.4^[54]^. We performed Mantel tests of isolation-by-distance (IBD) and isolation-by-environment (IBE) using the R package vegan, with significance determined based on 999 permutations.

### Demographic history inference

To estimate the origin and population divergence times of *L. oblata*, we reconstructed a time tree of Caprifoliaceae. Our analysis included 20 Caprifoliaceae samples, comprising seven population-representative *L. oblata* individuals. We selected one individual with the highest sequencing depth from each of the seven populations: M32 in JK, M61 in SS, M62 in BHS, M91 in JMS, M111 in BJS, M122 in WTS, and M165 in HDL. *Viburnum sargentii* Koehne (Viburnaceae) was chosen as the outgroup. Following the methodology outlined in the section 'Population genomic analyses', we constructed a maximum likelihood phylogenetic tree using IQ-TREE. Furthermore, a secondary time-calibrated divergence analysis was performed using the MCMCtree program from the PAML package v4.9^[55]^. The crown ages of both *Lonicera* L. (41.4–20.8 million years ago [Mya]) and Dipsacales (74.1–60.1 Mya) were set following Yang et al.^[56]^ and Li et al.^[57]^, respectively.

To infer the demographic history of *L. oblata*, we utilized Pairwise Sequentially Markovian Coalescent (PSMC) v0.6.5^[58]^ with default parameters. The sampling method was consistent with the above-mentioned phylogenetic analysis. In 2018, we reintroduced 52 seedlings, which were generated in 2016, to the Beijing Songshan National Nature Reserve. Of these, only eight individuals survived, and none of them flowered until 2025. Hence, we set the generation time of *L. oblata* as 10 years. We estimated the mutation rate to be 1.43e–8 per site per generation based on the *de novo* genome assembly of *L. oblata*. We further employed Stairway Plot 2^[59]^ to estimate recent effective population size (*N*e) via the folded site frequency spectrum (SFS) generated by ANGSD v0.921^[60]^.

We performed coalescent simulations with fastsimcoal2^[61]^ to identify the best-fitting demographic model based on 2D joint-folded SFS with easySFS^[62]^. Based on the results of the population structure conducted previously, we classified the seven populations into three lineages: southern (HDL), central (BJS and WTS), and northern (BHS, JK, JMS, and SS). We performed 100 independent runs for each model with 100,000 simulations (-n 100,000) and 40 conditional maximization (ECM) cycles (-L 40) for estimating the parameters. The best model was evaluated through the Akaike Information Criterion (AIC) across the 100 independent runs^[61]^. Confidence intervals for the parameters of the best model were obtained through 100 bootstrap replicates, with 50 independent runs conducted for each bootstrap.

We inferred gene flow events among populations using TreeMix v1.11^[63]^ based on the pruned SNPs dataset. Migration events (*m*) from 1 to 10 were performed with ten iterations each, and the parameters '-global -se -noss' were set to calculate standard errors and avoid overcorrection. The result files were used as input for the R package OptM v0.1.6^[64]^ to determine the optimal number of migration edges.

### Inbreeding and genetic load evaluation

We utilized VCFtools v1.9 to calculate whole-genome heterozygosity and the inbreeding coefficients (*F*_IS_) with the '-het' parameter. Subsequently, we employed PLINK v1.9 to identify long runs of homozygosity (ROH) across 140 individuals with the '--homozyg' parameter. ROH was defined as regions exceeding 1 kb in length, containing at least 50 SNPs, and devoid of any heterozygous loci. We categorized ROH into two groups: short (1–100 kb) and long (> 100 kb) according to Zhu et al.^[65]^. To quantify genomic inbreeding, we calculated *F*_ROH_, the fraction of the genome in ROH, by dividing the total length of all effective ROH within an individual by the genome length of *L. oblata.*

To estimate the genome-wide genetic load in *L. oblata*, we designated homozygous allele genotypes exceeding 50% (70 individuals) as the ancestral state^[65]^. To mitigate potential biases in downstream analyses arising from deleterious mutations in the reference genome, we excluded genomic sites that were inconsistent with the reference sequence from the high-quality SNPs dataset obtained in the section 'Whole-genome resequencing and variant calling'. After establishing a variant annotation database using SnpEff v5.1^[66]^ based on the *L. oblata* genome, we annotated SNPs in the 140 individuals and classified variants into three categories: synonymous (SYN), missense, and loss-of-function (LoF). We further categorized missense variants as nonsynonymous deleterious (DEL; SIFT score < 0.5) or tolerated (TOL; SIFT score ≥ 0.5) variants using SIFT 4G v6.2.1^[67]^, while excluding 'NA' and 'low confidence' sites. We counted the number of SYN, LoF, DEL, and TOL mutation sites among the total derived alleles. The population genetic load of *L. oblata* was quantified as the ratio of DEL and LoF mutations (including both heterozygous and homozygous sites) to SYN mutations. Finally, we assessed the strength of population purifying selection by computing the genetic diversity of 0-fold and 4-fold degenerate sites and their ratio (*π*_0_/*π*_4_) using the script get_degeneracy.py (https://github.com/hui-liu/Degeneracy).

### Selective sweep detection

To identify genomic signatures of local adaptation in *L. oblata*, we selected two populations for selective sweep analysis: HDL from the southernmost edge and JK from the northeasternmost edge. This selection was based on the results of population genetic structure, divergence time, and geographical distribution. Following Dong et al.^[68]^, we identified outlier regions indicative of potential selective sweeps as those positioned in the top 5% for both *F*_ST_ values and ln (*π* ratios). Candidate genes within these regions were functionally annotated via the GO database.

### Genotype-environment association analyses

We obtained and standardized 19 bioclimatic variables (bio1–bio19) from WorldClim (www.worldclim.org) across the period 1970–2000 at a spatial resolution of 2.5 arc-minutes. We identified key bioclimatic variables through the R package gradientForest^[69]^ and applied Pearson correlation filtering (|*r*| < 0.8) for subsequent analyses. We filtered the high-quality SNP data (obtained in the section 'Whole-genome resequencing and variant calling') with the parameter MISS > 0.99 to conduct genotype-environment association (GEA) analyses. Firstly, we used a univariate latent-factor linear mixed model (LFMM)^[70]^ implemented in the R package LEA^[71]^ across seven ancestral populations identified by Admixture to control population structure. Each environmental variable was analyzed through five independent runs, consisting of 5,000 burn-in and 10,000 sampling iterations. SNPs with *p*-values ≤ 1.0e–5 that were associated with at least one environmental variable were considered environment-associated SNPs^[70]^. Secondly, we performed redundancy analysis (RDA)^[72]^ in the R package vegan to detect multivariate associations between SNPs and the retained environmental variables. Significant environment-associated SNPs were identified as those exhibiting loadings in the tails of the distribution, utilizing a standard deviation cutoff of 3.5 along one or more RDA axes^[73]^. Finally, we identified core variants as those detected by both LFMM and RDA. We also performed analyses on the 15 soil variables, but obtained weak correlations, which were consequently excluded from the final GEA analyses.

### Genomic offset assessment

We predicted the adaptation of *L. oblata* populations to future climate change based on the contemporary genotype-environment relationships. The genomic offset was quantified as the Euclidean distance between current and projected genomic-climate spaces^[74]^, where a high value implies weak potential for climate adaptation. Spatial visualization of offset values was generated using ArcGIS v10.8. We extracted future climate data (2081–2100) for 19 bioclimatic variables at each sample location, and employed three models (ACCESS-CM2, BCC-CSM2-MR, and CMCC-ESM2). For each given model, we calculated the genomic offset using gradient forest under four shared socioeconomic pathways (ssp126, ssp245, ssp370, and ssp585) with the 19 environmental variables. Given the high cross-model correlations in genomic offset, the average values were used to enhance prediction robustness for assessing the potential of local adaptation under future climate change.

## Results

### Genome assembly and annotation

**Figure 1:**
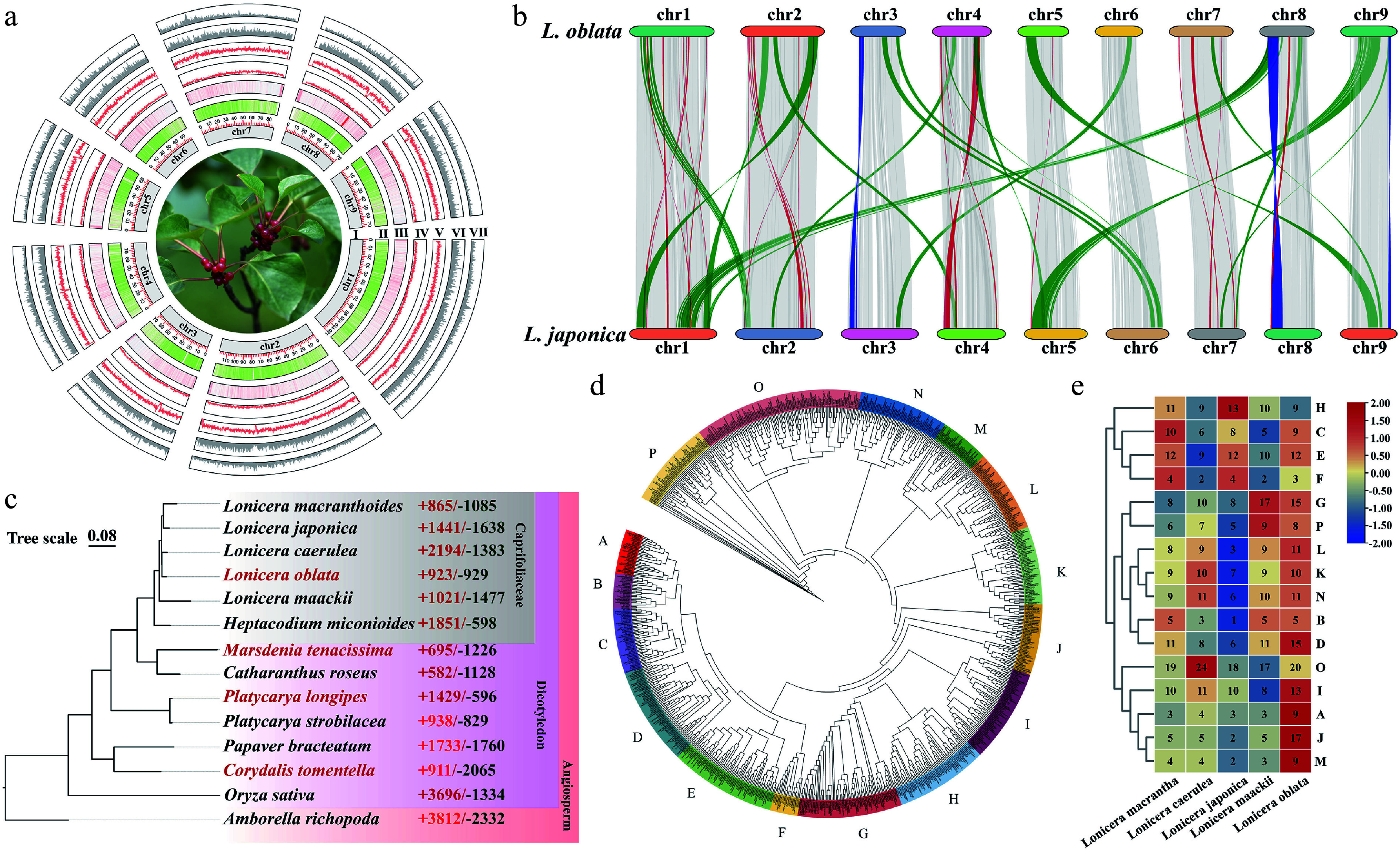
Genome assembly and comparative genomic analyses of *Lonicera oblata.* (a) Genomic characterization of *L. oblata*; (I) Chromosome, (II) Gene density, (III) GC content, (IV) Long terminal repeat retrotransposons (LTRs) content, (V) LTR-Gypsy content, (VI) LTR-Copia content, (VII) DNA transposons. (b) Collinearity analysis between *L. oblata* and *L. japonica*. The gray segments represent the gene collinearity, the blue segments represent chromosomal inversions, and the orange and red segments represent chromosomal translocations. (c) Phylogenetic tree of 14 species reconstructed from 238 single-copy orthologs. The samples with red Latin names are limestone-endemic species. The red and green numbers on the right indicate the number of orthologous groups that have expanded and contracted, respectively, compared to closely related species. (d) Phylogenetic tree of the bHLH family from *Lonicera*, constructed using the maximum-likelihood method (JTT + R6 model; 1,000 bootstrap replicates). The colored bands divide the bHLH family into 16 subfamilies. (e) Heatmap of the bHLH subfamilies in *Lonicera*; the numbers in the heatmap represent the number of gene subfamilies for each corresponding species. The red-blue gradient illustrates the variability of the number of subfamily genes.

We generated a chromosome-scale genome assembly of *L. oblata* by integrating multiple datasets, including Illumina short reads, PacBio Revio long reads, and Hi-C chromatin interaction data (Supplementary Tables S1, S2). The final assembly spans 786.92 Mb and has a GC content of 36.98% (Fig. 1a; Table 1). The assembly demonstrates high continuity (scaffold N50 = 78.49 Mb), completeness (average depth: 113.6 ×; coverage: 99.94%), and accuracy (97.80% of benchmarking universal single-copy orthologs were recovered) (Table 1). The Hi-C interaction matrix confirmed clear chromosomal compartmentalization, with 94.54% of sequences anchored to nine pseudo-chromosomes (Supplementary Fig. S1; Table 1). We identified 33,034 protein-coding genes with a mean length of 1,138.81 bp (Table 1). Of these, 65.34% and 35.55% of the genes were annotated by the GO and KEGG databases, respectively (Supplementary Table S3). We identified repetitive sequences that comprise 66.47% of the genome, with LTR retrotransposons representing the dominant class at 45.36%. Among the LTR families, LTR-Gypsy (18.94%) and LTR-Copia (10.19%) elements were the most abundant, collectively contributing over 29% to the genome (Supplementary Table S3). These LTRs have gradually accumulated in the genome over the past 40 Mya (calculated based on *Ks* values of LTR sequences), with the peak of their emergence occurring approximately 1.34 Mya (Supplementary Fig. S2a).

**Table 1:** Summary of the assembly and annotation of the *Lonicera oblata* genome.

Item	Statistic
Genome size (Mb)	786.92
GC content (%)	36.98
Contig N50 (bp)	78,486,388
Contig N90 (bp)	58,410,856
Mapping rate (%)	99.65
Complete BUSCOs (%)	97.80
Average depth (×)	113.62
Coverage (%)	99.94
Total number of genes	33,343
Average CDS length (bp)	1,138.81

Comparisons among the genomes of six Caprifoliaceae species revealed significant variation in genome size, ranging from 635 to 904 Mb. *L. oblata* (endemic to NCM; 787 Mb) and *L. japonica* (a globally invasive species; 904 Mb) exhibited a 14.87% difference in genome size (Supplementary Table S4). We observed notable chromosomal positioning and translocation between *L. oblata* and *L. japonica*, particularly on Chr3 and Chr8 (Fig. 1b; Supplementary Fig. S3).

### Comparative genomic analyses

To decipher the evolutionary dynamics and potential environmental adaptation strategies of *L. oblata*, we conducted a *Ks* distribution analysis, revealing a conserved WGD event shared by the Caprifoliaceae (Supplementary Fig. S2b). WGD accounted for over 35% of gene family expansions within Caprifoliaceae (10,041/33,343 in *L. oblata*; 10,642/33,939 in *L. japonica*; 11,117/32,075 in *H. miconioides*), while tandem duplications contributed to approximately 10% (Supplementary Fig. S4). A total of 451,741 protein-coding genes from 14 species were classified into 22,990 orthogroups using OrthoFinder. Among these, 6,663 conserved orthogroups were identified across the 14 angiosperm species, while Caprifoliaceae and *Lonicera* shared 102 and 25 conserved orthogroups, respectively (Supplementary Fig. S2c; Supplementary Table S5). We identified 2,227 orthogroups shared by four limestone-endemic species (Supplementary Fig. S2d). Functional profiling demonstrated significant enrichment (FDR < 0.05) in three critical domains: photosynthetic and energy transduction mechanisms (GO:0022900: electron transport chain, GO:0009772: photosynthetic electron transport, and GO:0019684: photosynthesis, light reaction), cation binding (GO:0030145: manganese ion binding and GO:0046914: transition metal ion binding), and root system development (GO:0022622) (Supplementary Fig. S5). KEGG pathway analysis confirmed coordinated adaptation through energy metabolism (ko00190: oxidative phosphorylation and ko00195: photosynthesis), replication and repair (ko03030: DNA replication, ko03420: nucleotide excision repair, and ko03440: homologous recombination), and flavonoid biosynthesis (ko00941) (Supplementary Fig. S5). These molecular signatures collectively delineate a sophisticated adaptive framework that combines photoprotection efficiency, reactive oxygen species scavenging capacity, and metabolic plasticity, which are essential for colonizing oligotrophic lithospheric niches.

The genome of *L*. *oblata* exhibited a significantly lower level of orthogroup expansion (923) compared to the highest number of 2,194 observed in *L*. *caerulea*, which thrives in a relatively cold habitat. Additionally, *L*. *oblata* demonstrated a lower level of orthogroup contraction (929) than the globally invasive species *L*. *japonica* (1,638). These findings among the five honeysuckle species further suggest divergent environmental adaptation strategies within the genus (Fig. 1c). GO annotation revealed that the genes associated with expansion in *L*. *oblata* were enriched in three biological processes: molecular functions (oxidoreductase activity), cellular processes (cell wall biosynthesis/metabolism), and transport mechanisms (Supplementary Fig. S6). Furthermore, KEGG pathway analysis identified core metabolic adaptations, including energy transduction (ko00195), photosynthetic efficiency (ko00196), phytohormone signaling (ko04075), and specialized metabolism (phenylpropanoid biosynthesis, ko00940) (Supplementary Fig. S6).

### Transcriptomic-metabolomic association analyses

PCA showed clear clustering of transcriptome samples, indicating good reproducibility among biological replicates (Supplementary Fig. S7a). Differential expression analysis across five pairwise comparisons identified 9,408 DEGs (after removing duplicates), including 11,538 upregulated and 7,186 downregulated genes (Supplementary Fig. S7b). Approximately 30% of the expanded genes in *Lonicera* and those shared with limestone-endemic species were differentially expressed under calcium stress conditions (Supplementary Fig. S7c).

We identified 249 and 166 putative transcription factors among the expanded orthogroups of *L. oblata* and the other three lithophytic species, respectively. Among these, the bHLH, TCP, and NAC families, which are involved in plant growth and responses to limestone environments, were notably enriched (Supplementary Table S6). We also identified these three families in the five *Lonicera* species and found that the bHLH family in *L. oblata* was significantly expanded within the genus, comprising 176 members (Supplementary Fig. S8; Supplementary Table S7). The phylogenetic tree of the bHLH family in *Lonicera* resolved 16 distinct subfamilies (A–P) (Fig. 1d). Notably, *L. oblata* exhibited the most significant expansion, particularly in subfamilies A, I, J, and M (Fig. 1e). Approximately 64.77% of the *bHLH* genes (114 out of 176) were derived from WGD/segmental duplication, a significantly higher proportion than in the other four *Lonicera* species (44.36%–53.70%) (Supplementary Fig. S9). Additionally, the number of tandemly duplicated genes in the bHLH family of *L. oblata* was greater than that in other congeneric species. These tandemly duplicated genes were often physically clustered on chromosomes (Supplementary Figs S10, S11), suggesting that tandem duplication contributed to the formation of gene clusters within these transcription factor families.

Under high-calcium stress, 88 of the 176 *bHLH* genes were expressed (FPKM > 1) (Supplementary Fig. S12). In the notably expanded subfamilies A and M, most members were induced by the treatment. Notably, *LoblChr1G00045910* of the subfamily A showed the highest expression level. Based on their expression patterns, the responsive *bHLH* genes were categorized into two clusters. Genes in cluster B displayed distinct expression peaks at various time points following calcium stress, implying a positive role in the stress response. *LoblChr1G00045910* and *LoblChr2G00065320*, shared with limestone-endemic species, exhibited the highest expression levels in the entire bHLH family, indicating that they may be key members involved in the calcium stress response in *L. oblata*.

**Figure 2:**
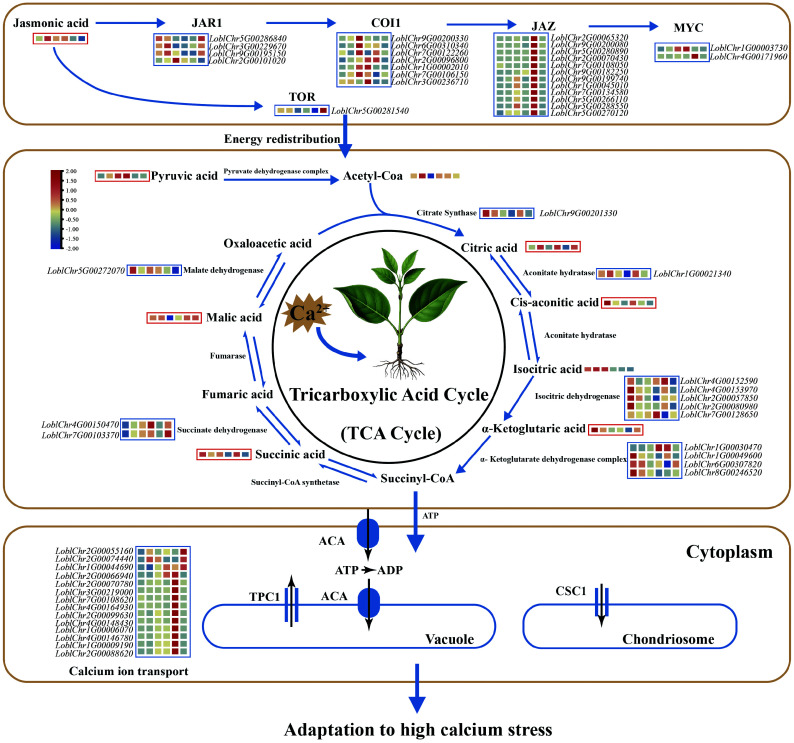
Mechanism of high Ca^2+^ adaptation in *Lonicera oblata*. The blue box represents the expression of the differentially expressed genes, while the red box reflects the abundance of differentially accumulated metabolites. The colored blocks within each box are arranged (left to right) in chronological order corresponding to the following time points: control (CK), 1, 3, 6, 12, and 24 h. Calcium stress induced pronounced changes in jasmonic acid (JA) signaling and *TOR* responses, with rapid induction of *JAR1* (peaking at 1 h), overall upregulation of *COI1*, strong induction of multiple *JAZ* genes during early to mid-stages, and significant activation of *MYC2/3* transcription factors, whereas TOR expression was reduced from 1 to 12 h. In the tricarboxylic acid (TCA) cycle, pyruvic acid increased from 1 h onward, citric acid accumulated at 1 and 6 h, while cis-aconitic acid and *α*-ketoglutaric acid decreased at early time points. Downstream intermediates, including succinic acid and malic acid, accumulated during 6–12 h and partially recovered by 24 h. The genes encoding aconitate hydratase and isocitrate dehydrogenase were differentially expressed at early stages, whereas fumarase, succinate dehydrogenase, and malate dehydrogenase genes showed higher expression at later stages. Most calcium ion transporter genes were differentially expressed, with expression levels peaking around 12 h. Calcium ion homeostasis under stress is achieved through JA-mediated organic acid chelation and active transport mechanisms.

In the metabolomic profiling, we detected a total of 1,636 metabolites across both ionization modes, with lipids, organoheterocyclic compounds, and organic acids each constituting over 15% of the identified compounds (Supplementary Fig. S13a). We identified 289 DAMs, including the phytohormone jasmonic acid (JA). JA levels increased markedly from 1 to 12 h (Supplementary Fig. S13b). *JAR1* was rapidly induced, peaking at 1 h; *COI1* showed sustained upregulation; multiple *JAZ* genes were strongly induced during early to mid-stages; and *MYC2/3* transcription factors were significantly activated. In contrast, *TOR* expression was consistently suppressed from 1 to 12 h (Fig. 2).

Joint KEGG annotation of DAMs and differentially expressed genes (DEGs) revealed co-enrichment in pathways including amino acid metabolism, the TCA cycle, and ascorbate metabolism (Supplementary Fig. S13c). Focusing on the TCA cycle, we found that pyruvic acid accumulated from 1 h onward, citric acid rose at 1 and 6 h, while *cis*-aconitic and *α*-ketoglutaric acids decreased at early time points. Downstream intermediates like succinic and malic acids accumulated during 6–12 h, partially recovering by 24 h. Consistent with these metabolic changes, transcript levels of genes encoding aconitate hydratase and isocitrate dehydrogenase were differentially expressed at early stages, while fumarase, succinate dehydrogenase, and malate dehydrogenase genes showed higher expression at later stages, coinciding with metabolite accumulation (Fig. 2). Additionally, the expression of most calcium ion transporter genes peaked around 12 h after stress (Fig. 2).

### Population structure and genetic diversity analyses

**Figure 3:**
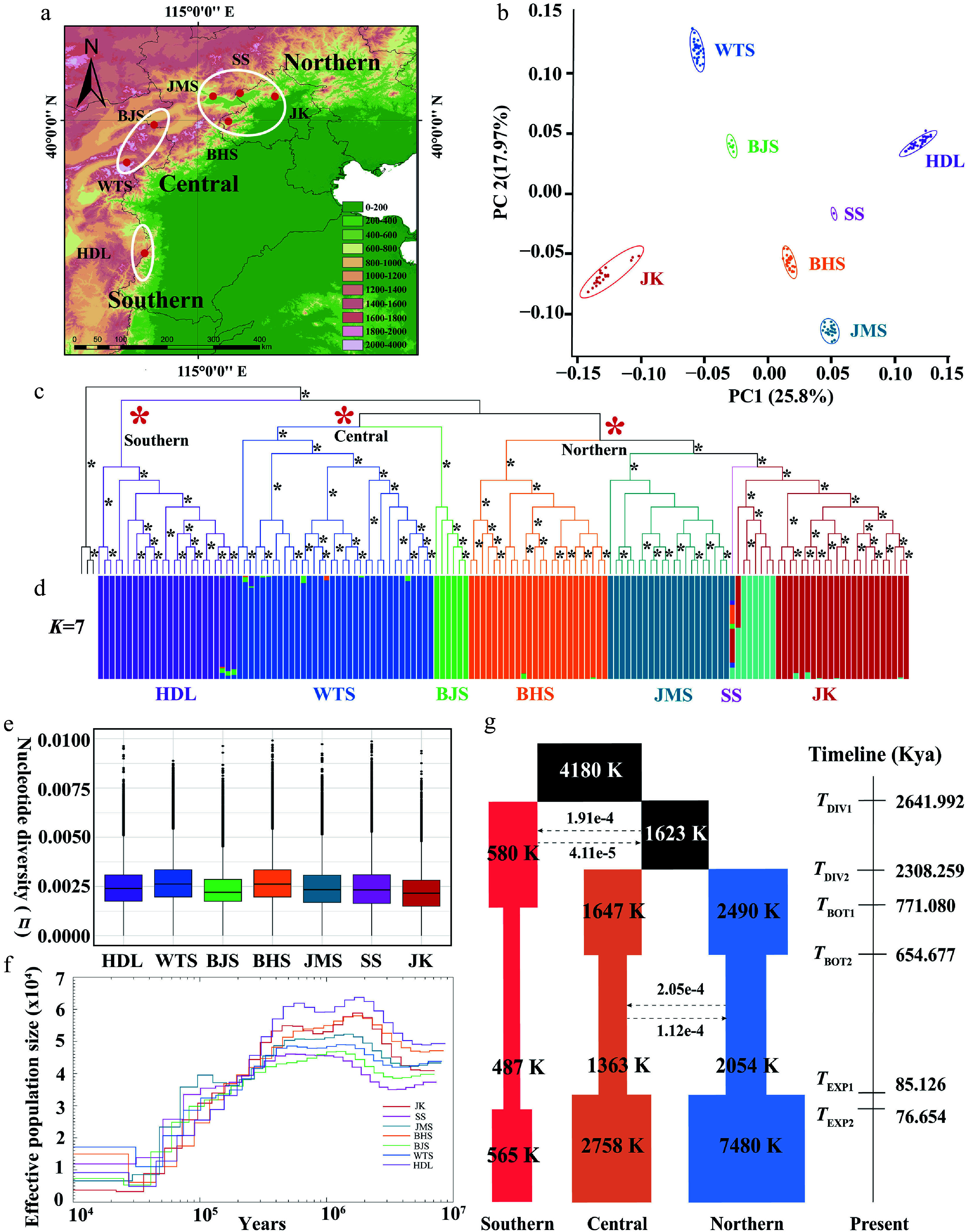
Distribution pattern, population structure, genetic diversity, and demographic history of *Lonicera oblata*. (a) Geographic distribution of the seven populations. (b) PCA plot of *L. oblata* populations. (c) A maximum likelihood phylogenetic tree of 140 *L. oblata* individuals, with *L. webbiana* as the outgroup; branches with full support (ultrafast bootstrap value = 100) are marked with *. (d) Population structure of *L. oblata* inferred by Admixture (optimal *K* = 7). (e) Nucleotide diversity (*π*) among the seven populations. (f) Pairwise Sequentially Markovian Coalescent (PSMC) modeling analysis of the seven populations of *L. oblata*, with a generation time of 10 years and a mutation rate (μ) of 1.43e–8 per site per generation. (g) Maximum likelihood estimation of the best-fit model (model 11) in fastsimcoal2 (see Supplementary Table S14). (a) The map is from the National Platform for Common Geospatial Information Services (Tianditu), with review number GS(2024)0650. Source: www.tianditu.gov.cn.

We obtained whole-genome resequencing data of 140 individuals for the seven populations (Fig. 3a; Supplementary Table S8). Clean reads were obtained after filtering (1.45 Tb; Supplementary Table S9), with an average mapping rate of 92.19% and a sequencing depth of 13.23 × (Supplementary Table S9). A dataset with 36,902,393 raw SNP was generated by GATK, and a high-quality data matrix with 9,586,400 SNPs was obtained for evaluating population genetic diversity and genetic load. Finally, a dataset of 1,892,302 LD-pruned SNPs was constructed for population genetic structure. The seven geographical populations were recovered in PCA (Fig. 3b), population phylogeny (Fig. 3c), and population structure (Fig. 3d; Supplementary Fig. S14a, b). The Admixture analysis performed across a range of *K* = 2–8 consistently demonstrated a robust genetic structure (Supplementary Fig. S14b). Though the optimal *K* was 7 (Supplementary Fig. S14a), three main lineages were classified into the southern lineage (HDL population), the central lineage (WTS and BJS populations), and the northern lineage (BHS, JMS, SS, and JK populations) (Fig. 3c). This three-lineage division was congruent with the major clades presented by the population phylogenetic tree (Fig. 3c). At higher *K* values (e.g., *K* = 7 and *K* = 8), fine-scale substructure was further detected within the JK population (Fig. 3d). The Mantel test demonstrated significant IBD (*r* = 0.452, *p* < 0.033, 999 permutations; Supplementary Fig. S14c) rather than IBE (*r* = 0.270, *p* < 0.867, 999 permutations; Supplementary Fig. S14d).

*F*_ST_ values varied significantly among the seven populations, ranging from 0.04 (SS and JMS) to 0.26 (JK and BJS) (Supplementary Table S10). It is worth noting that pairwise differentiation was higher than 0.11 among population pairs, excluding the SS population, which only has one individual. Nucleotide diversity varied from 2.24e–3 (JK) to 2.80e–3 (WTS) (Fig. 3e; Supplementary Table S11). We identified more than 20,000 private alleles in the HDL, WTS, and JK populations, while each of the remaining populations had fewer than 7,000 private alleles (Supplementary Table S11). LD decay patterns revealed divergent evolutionary trajectories: WTS and BHS populations exhibited rapid LD decay at 3.63 kb and 6.54 kb (*r*^2^ = 0.2), consistent with their higher genetic diversity. In contrast, JK (20.40 kb, *r*^2^ = 0.2) and BJS populations (14.79 kb, *r*^2^ = 0.4) demonstrated slow LD decay (Supplementary Fig. S15a; Supplementary Table S12).

### Demographic history inference

The crown age of *L. oblata* was estimated at 2.59 Mya (HPD: 3.68–1.61 Mya, Supplementary Fig. S16a), with subsequent northward migration and divergence during 1.97–0.95 Mya (Supplementary Fig. S16a). The PSMC analysis revealed a significant population decline since ca. 200 Kya. Stairway Plot 2 analysis indicated that five populations demonstrated sustained *N*e expansion since ca. 100 Kya. This expansion was linked to the warmer climatic conditions and habitat expansion during the Last Interglacial Marine Isotope Stage 5^[75]^, with population size then stabilizing during the Holocene (Supplementary Fig. S15b). However, the BJS population exhibited a continuous decline from ca. 100 to 1 Kya (Supplementary Fig. S15b), ultimately reaching the lowest contemporary *N*e among all populations.

We utilized fastsimcoal2 to evaluate gene flow and population demography. A total of 16 alternative demographic models were tested, and model 11 was identified as the best-fitting model, which demonstrated the lowest AIC (Fig. 3g; Supplementary Fig. S17a, S17b; Supplementary Table S13). According to model 11, the initial divergence began at 2.64 Mya, followed by the divergence of the central and northern lineages at 2.31 Mya (Fig. 3g). A low level of ancient gene flow between the southern lineage and the common ancestor of the central and northern lineages was detected (Fig. 3g; Supplementary Table S14). In contrast, we observed a high level of gene flow between the central and northern lineages (Fig. 3g). Gene flow was also detected between HDL (southern) and JK (northern) as analyzed by TreeMix (Supplementary Fig. S16b–S16d). In addition, all three lineages experienced nearly synchronous population contractions and expansions during the period of 743–77 Kya (Fig. 3g).

### Inbreeding and genetic load analyses

**Figure 4:**
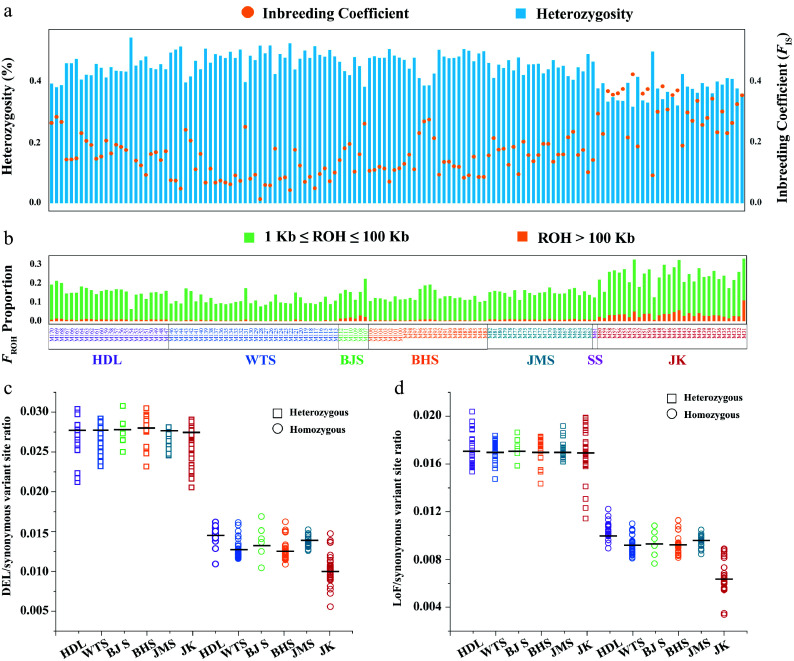
Analyses of inbreeding and genetic load. (a) Per-site genome-wide heterozygosity is represented by bar plots in the upper panel (left axis), while the inbreeding coefficient is indicated by orange circles in the upper panel (right axis). (b) *F*_ROH_ proportion among populations. The colors illustrate the summed lengths of short (1 kb ≤ ROH ≤ 100 kb, green) and long (ROH > 100 kb, orange) ROH per individual. Samples are arranged according to the geographical distribution pattern of *L. oblata* from South to North. (c) The ratio of derived deleterious (DEL) variants and (d) Loss of function (LoF) variants to derived synonymous variants for heterozygous (rectangles) and homozygous (circles) tracts per individual among *Lonicera oblata* populations.

We calculated the individual-level *F*_ROH_, genomic heterozygosity, and *F*_IS_ of *L. oblata* (Fig. 4a, b; Supplementary Table S15), revealing a negative correlation between inbreeding levels and genomic heterozygosity. The JK population exhibited the highest proportion of long ROH (> 100 kb), inbreeding level (*F*_IS_ = 0.30), and the greatest *F*_ROH_ (0.24) (Supplementary Fig. S18a, S18c; Supplementary Table S15), yet it had the lowest heterozygosity (0.19; Supplementary Fig. S18b). In contrast, the WTS population showed the lowest *F*_ROH_ (0.11; Supplementary Fig. S18a; Supplementary Table S15) and *F*_IS_ (0.10; Supplementary Fig. S18c; Supplementary Table S15), while exhibiting the highest heterozygosity (0.25; Supplementary Fig. S18b; Supplementary Table S15).

Genetic load analyses revealed similar values for the ratio of DEL/synonymous variant sites among the six populations in the heterozygous state, with an average of 2.68e–2 (Fig. 4c; Supplementary Table S16). The ratio of LoF/synonymous variant sites showed a comparable average of 1.72e–2 (Fig. 4d; Supplementary Table S16). However, the DEL and LoF ratios varied in the homozygous state among populations (Fig. 4c, d; Supplementary Table S16). Specifically, the southernmost population (HDL) exhibited the highest levels of genetic load for both homozygous DEL (1.45e–2) and LoF (1.04e–2) mutations, while the northeasternmost (JK), which has sub-population structure, displayed the lowest levels of genetic load for both homozygous DEL (1.03e-2) and LoF (6.46e–3) mutations (Fig. 4c, d; Supplementary Table S16). Furthermore, the lowest ratio of *π*_0_/*π*_4_ was identified in the JK population (1.42; Supplementary Fig. S19), indicating that this population experienced the strongest purifying selection.

### Genome-wide selective sweeps analysis

**Figure 5:**
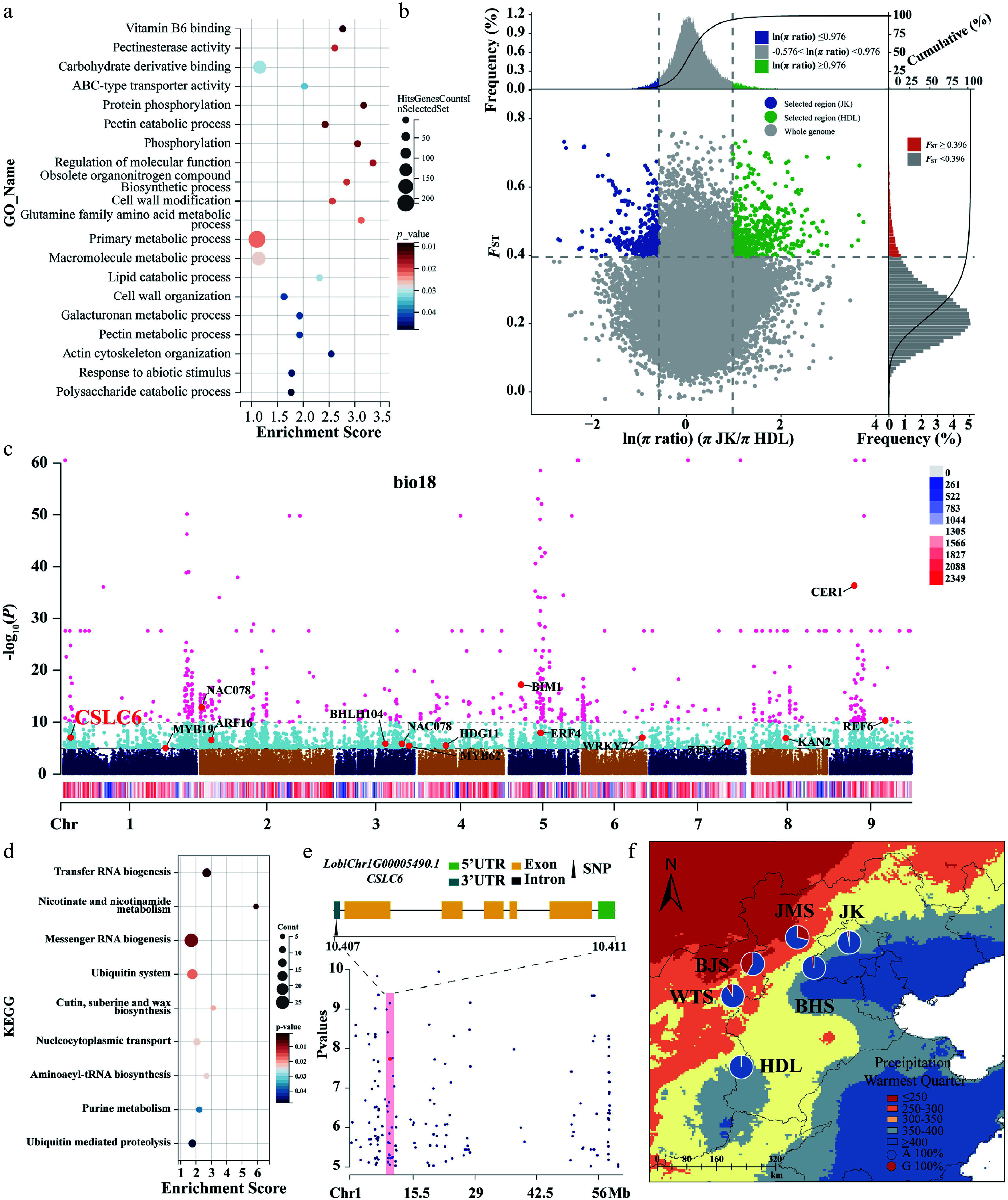
Selective sweeping analyses for the Northern JK and Southern HDL populations, along with genome-wide variation association analyses of *Lonicera oblata*. (a) GO enrichment results for genes containing selective candidate SNPs. The size and color of the circles represent the number of genes enriched in specific pathways and the degree of pathway enrichment, respectively. (b) Distribution of ln (*π* ratios) and *F*_ST_ values calculated in 100-kb windows with 10-kb steps. (c) The Manhattan plot of SNPs associated with the environmental variable bio18 across the entire genome estimated with LFMM. The vertical axis represents the *p*-value of the relationship between SNPs and bio18, ranging from 1 to 1.0e–60. The light blue and pink dots indicate the threshold *p*-value of 1.0e–5 and 1.0e–10, respectively. The colored strips with a red-blue gradient represent the density of SNPs on the chromosome. (d) KEGG enrichment results for genes containing selective candidate SNPs. (e) The location of the genes where the core SNPs are situated on Chr1. The black triangle indicates that the adaptive SNP is in the 3' UTR region of the gene. The red dots and shaded areas highlight the positions of the target SNPs within the locally magnified section of the Manhattan plot. (f) The genotype frequency distribution of core SNPs corresponding to precipitation gradients across different populations. Blue represents genotype A, while orange represents genotype G. The red and blue areas on the map indicate precipitation levels during the hottest season (bio18).

We identified 1,327 candidate selective regions and 1,217 selected genes (Fig. 5a; Supplementary Fig. S20). Chromosomal mapping revealed Chr8 as the primary selection hotspot, containing 24.1% of all identified regions (*n* = 320), with other clusters distributed across the remaining chromosomes (Supplementary Fig. S20). GO enrichment analysis of the selected genes demonstrated significant enrichment in three functional pathways: cell wall modification and organization (GO:0042545, GO:0071555, GO:0045490, GO:0030036, etc.), ABC-type transporter activity (GO:0140359), and the glutamine family amino acid metabolic process (GO:0009064) (Fig. 5b). We observed pronounced selection signals in genes associated with cell wall modification and organization, particularly in pectin methylesterases (*PMEs*) and xyloglucan endotransglucosylases/hydrolases (*XTHs*). These functionally significant enzymes play a crucial role in modulating cell wall composition, with the majority of *PMEs* and *XTH* genes being localized to Chr8 (Supplementary Fig. S20), suggesting potential adaptive advantages for abiotic stress tolerance in *L. oblata*.

### Genomic variants associated with local adaptation

We identified three bioclimatic variables (bio7, bio18, and bio19) for GEA analyses (Supplementary Fig. S21; Supplementary Table S17). Using LFMM, we obtained a total of 6,274 SNPs after excluding the overlapping sites, including 3,454 associated with bio7, 1,878 with bio18, and 1,772 with bio19 (Supplementary Fig. S22). These SNPs were widely distributed across the whole genome rather than clustered in specific genomic regions, with overlaps among the environmental variables (Fig. 5c; Supplementary Table S18). Subsequent enrichment analyses of the genes harboring these SNPs revealed variable-specific metabolic pathways. The genes associated with bio7 demonstrated significant enrichment in phenylalanine/tyrosine/tryptophan biosynthesis (ko00400) and alanine/aspartate/glutamate metabolism pathways (ko00250) (Supplementary Fig. S23a), while those related to bio19 were overrepresented in thiamine metabolism (ko00730) (Supplementary Fig. S24a). Notably, the genes associated with bio18 showed significant enrichment in cutin, suberin, and wax biosynthesis pathways (ko00073), highlighting distinct biochemical adaptation strategies corresponding to different environmental pressures (Fig. 5d). Further characterization revealed strong selection signals for transcription factors involved in environmental adaptation, including *bHLH104*, *NAC78*, and *NAC100*, as well as the developmental regulators *TCP5* and *TCP12*. Notably, *bHLH104* emerged as a core regulatory element demonstrating convergent selection across all three environmental variables (Fig. 5c; Supplementary Figs S23b, S24b). The first two of the three RDA axes accounted for 75.52% of the variance (Supplementary Fig. S25). RDA identified 8,695 SNPs showing significant associations with the three bioclimatic variables (Supplementary Figs S26, S27).

Finally, we identified 1,286 core variants through the overlap of LFMM and RDA. These variants were primarily located on Chr1, Chr2, Chr6 and Chr8 (Supplementary Table S18), which we classified as core potentially adaptive variants for local adaptation. We performed statistical analyses of genotype frequencies for these SNPs, which exhibited strong genotype-environment correlations (Supplementary Tables S19, S20). Notably, SNP15527 on Chr1 demonstrated significant geographical differentiation. It is located within the 3' UTR of *LoblChr1G00005490.1* (Fig. 5e), a homolog of *AtCSLC6* encoding a xyloglucan synthase essential for cell wall biosynthesis. Spatial distribution analysis revealed a pronounced allele frequency cline corresponding to precipitation gradients: the A allele predominated in populations located in the high-precipitation eastern mountain regions, while the G allele was primarily found in populations from the arid northwestern territories (Fig. 5f). This geographical pattern mirrors the differential selection pressures imposed by contrasting hydrological regimes, suggesting the functional significance of xyloglucan metabolism in environmental adaptation.

### Genomic offset prediction for future climate change

**Figure 6:**
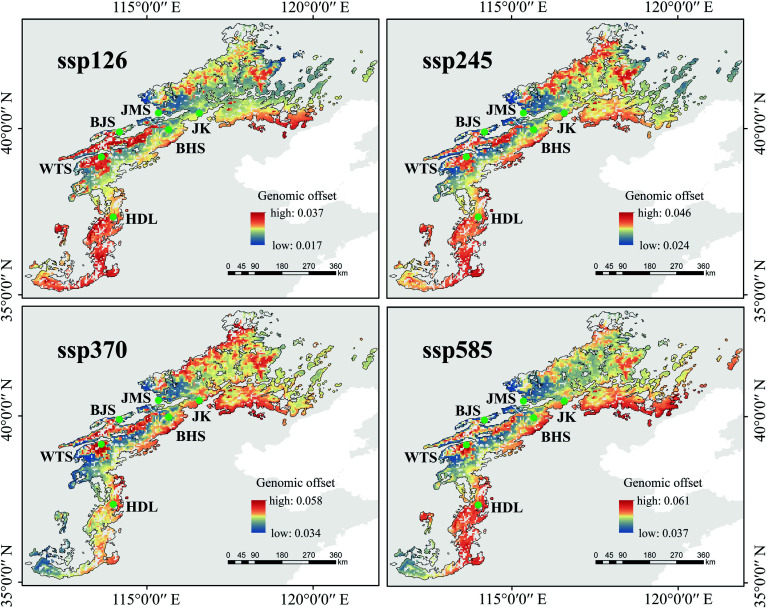
Predicted genomic offset of *Lonicera oblata* in response to future climate change (2081–2100) using the BCC-CSM2-MR model. Source: the National Platform for Common Geospatial Information Services (Tianditu), with review number GS(2024)0650. www.tianditu.gov.cn

We performed long-term predictions of population genomic offset from 2081 to 2100 under three models (ACCESS-CM2, BCC-CSM2-MR, and CMCC-ESM2). As carbon dioxide emissions increase, the overall genomic offset values gradually increased across the three models (Fig. 6; Supplementary Fig. S28). All models exhibited a northward-increasing trend in climate change adaptation across the lineages, indicating that the northern Taihang Mountains are more favorable for the survival of *L. oblata* under future climate change. Our primary focus was on the genomic offset patterns under the BCC-CSM2-MR model (Fig. 6), which aligned more closely with the climate change projections for NCM^[74]^. Specifically, the BJS and JMS populations demonstrated the lowest values of genomic offset (e.g., 4.22e–2 and 4.50e–2 under ssp585; see Supplementary Fig. S29 and Supplementary Table S21 for detailed genomic offset values under different ssp scenarios). Under ssp585, the southern lineage (HDL) showed the highest genomic offset value of 5.25–2, followed by the central lineage (4.72e–2), while the northern lineage displayed the lowest value (4.67e–2) (Supplementary Table S21).

## Discussion

### Genomic footprints of local adaptation to limestone habitats

We presented the first chromosome-level genome of *L. oblata*, a rare sky island species endemic to the NCM, and elucidated its genomic adaptation to limestone habitats. Notably, the *L. oblata* genome exhibited the highest repetitive sequence content (66.47%) among the five *Lonicera* species, yet maintained a medium genome size (Supplementary Table S4). Elevated repetitive content may drive speciation and environmental adaptation^[76−78]^. LTRs are key drivers of genome evolution, speciation, and local adaptation^[79−81]^. They are abundant in *L. oblata* (45.36%), suggesting a potential for adaptation to limestone. Furthermore, we observed a significant expansion of stress-tolerance-related transcription factor families (e.g., bHLH, TCP, and NAC). The bHLH family mediates stress responses such as salt tolerance (*NtbHLH123*) and drought adaptation (*MdbHLH130*)^[82,83]^. The expansion of the bHLH family in *L. oblata* (176 members vs an average of 136 in *Lonicera*; Supplementary Fig. S8) combined with the marked upregulation of these genes under calcium stress (Supplementary Fig. S12) collectively demonstrates the family's key role in adaptation to limestone-specific abiotic stresses. We also identified expanded orthogroups associated with photosynthesis, electron transport, and DNA repair (Supplementary Figs S5, S6), indicating an adaptive optimization of photoprotection and resistance to oxidative stress. Moreover, we detected extensive structural variations in the genomes of the locally endemic *L. oblata* and the globally invasive *L. japonica*. Chromosomal translocations and rearrangements may enhance environmental adaptation by altering gene order, exposing regulatory elements, or stabilizing adaptive allele complexes through recombination suppression^[84,85]^. The significant segmental translocation identified between these two *Lonicera* species implies its potential contribution to the local adaptation of *L. oblata*.

Our transcriptomic and metabolomic analyses under calcium stress revealed that *L. oblata* rapidly activates jasmonic acid signaling while suppressing TOR expression, redirecting resources toward stress tolerance (Fig. 2). This hormonal reprogramming is coupled with enhanced TCA activity and upregulation of calcium transporter genes, facilitating calcium detoxification (especially during the rainy season) through organic acid chelation and active transport. However, these responses rely on sustained energy supply^[86,87]^. Unlike the papery leaves of other congeneric species, *L. oblata* exhibits leathery and thickened foliage^[88]^. Although this entails a higher energetic cost and lower photosynthetic efficiency for plants^[89,90]^, it provides support for sustained carbon assimilation and energy provision. Therefore, these morphological and metabolic traits collectively represent a crucial adaptive strategy that enables its exclusive distribution in the open, high-calcium limestone environments of mountain summits.

### Divergent levels of inbreeding and genetic load

Severe inbreeding commonly occurs in small and isolated populations. Without intervention, it can create a vicious cycle of inbreeding depression, ultimately compromising population viability^[91−93]^. Long ROH implies recent inbreeding and potential accumulation of genetic load, whereas short ROH reflects historical inbreeding^[94,95]^. ROH length thresholds are generally shorter in plants^[65,96]^ compared to animals^[97,98]^.

Strikingly, the northeasternmost and youngest population (JK) exhibited the highest inbreeding level, yet the lowest genetic load. In contrast, the southernmost and oldest population (HDL) showed moderate inbreeding but the highest genetic load. This paradox may be explained by the intense inbreeding in JK, which likely intensified purifying selection. This process purges homozygous DEL/LoF mutations, thereby reducing genetic load in JK. Similar scenarios of high inbreeding associated with low genetic load have been reported in other endangered plants (e.g., *Ostrya rehderiana* Chun^[99]^; *Cupressus gigantea* W. C. Cheng & L. K. Fu^[100]^) and animals (e.g., saola^[97]^, snow leopards^[98]^). The harsh yet non-urgent characteristics in these cases imply that the purging of deleterious mutations is one of the vital mechanisms by which small populations maintain viability. However, this genetic stability in JK represents a fragile balance. Although intense purifying selection removed deleterious alleles and maintained short-term viability, it simultaneously resulted in a loss of genetic diversity, as evidenced by JK's low nucleotide diversity (2.24e–3; Fig. 3e), strong sub-population^[19]^ structure (Fig. 3d), and the lowest heterozygosity (0.19; Supplementary Fig. S18b) among all populations. Such severe erosion of genetic variation might constrain the population's long-term adaptive potential, rendering it vulnerable to future environmental change^[99]^.

### Genomic variants associated with local adaptation

Populations of the same species can exhibit divergent responses to climate change due to local adaptation to heterogeneous environments^[73]^. Our GEA analyses identified 1,286 core SNPs highly correlated with environmental variables. For instance, SNP15527, located within gene *LoblChr1G00005490.1*, is homologous to *AtCSLC6*, which potentially influences hemicellulose synthesis and cell wall integrity^[101,102]^. Hemicelluloses intertwine with the cellulose network to provide mechanical support, whereas the pectin matrix maintains cell wall hydration and plasticity^[103]^. Drought and elevated CO_2_ reduce cell wall pectin and hemicellulose content^[104]^. Notably, the BJS, JMS, and WTS populations harbored higher frequencies of the G allele at SNP15527 (Fig. 5f), suggesting stronger potential for drought adaptation relative to the other populations. We speculate that, under drought and heat stress, individuals carrying the G allele might adjust cell wall composition to maintain turgor pressure and prevent tissue collapse more effectively, thereby conferring a selective advantage. Projected global warming may accelerate divergence in allele frequencies for cell wall-related genes among populations, thereby intensifying the divergent evolution of local adaptation and viability. Organisms can adapt to rapid climate change through subtle shifts in polygenic allele frequencies^[73]^. We propose that these adaptive genetic variations underlie the local adaptation of *L. oblata* to the heterogeneous environments of the NCM.

### Genomic vulnerability under climate change and conservation priorities

Population genomics provides a robust, quantitative, and comparable foundation for the conservation, assessment, and management of regional biodiversity^[105]^. In this study, the southern lineage (HDL) displayed a significant adaptive lag under SSP585 (Fig. 6), indicating its weak adaptation to future climate change. Moreover, this lineage harbored the highest number of private alleles (25,551; Supplementary Table S11) and represented a distinct evolutionary unit (Fig. 3c; Supplementary Fig. S14b). The fastsimcoal2 analysis (Fig. 3g) identified ancestral gene flow from the southern lineage to the ancestor of the central and northern lineages, which was corroborated by TreeMix results (Supplementary Fig. S16b−d). This evidence establishes HDL as an ancient genetic source for other populations. Therefore, prioritizing *in situ* conservation for the HDL population is vital to preserve its unique genetic diversity and ancestral gene pool, which is crucial for the species' long-term adaptive potential.

In contrast, the central and northern lineages that cover a large distribution range exhibited lower genomic offset values (Fig. 6), suggesting relatively favorable environments in the northern Taihang Mountains and the intersection of the Taihang and Yanshan Mountains. Fastsimcoal2 (Fig. 3g) also revealed gene flow between the central and northern lineages. Considering their vast suitability range^[19]^, potentially large population size, and close phylogenetic relationships, we recommend both *in situ* and *ex situ* conservation for populations within this region (excluding JK). However, the northeasternmost JK population maintains a delicate genetic equilibrium, characterized by high inbreeding (*F*_IS_ = 0.30; Supplementary Fig. S18a) coupled with low genetic load (homozygous DEL: 1.03 × 10^−2^, LoF: 6.46 × 10^−3^; Fig. 4c, d). Given its unique evolutionary history and fragile genetic structure (Fig. 3d), we advocate for *in situ* conservation that precludes assisted gene flow from other populations, so as to avoid disturbing the balance of such a genetically fragile population.

## Conclusions

We generated a chromosome-level genome assembly for the endangered sky island shrub *L. oblata* and re-sequenced 140 individuals across its distribution range in the NCM. Comparative genomics revealed a high repetitive content and a significant expansion of stress-responsive transcription factors, which were corroborated by transcriptomic analysis (e.g., bHLH family), likely underpinning limestone adaptation. Despite low genetic diversity and high inbreeding, purifying selection likely reduced the genetic load in the youngest population (JK), highlighting its potential for small population persistence. Landscape genomics identified 1,286 core candidate adaptive SNPs and quantified genomic offset, enabling population-specific conservation strategies. This study enhances our understanding of local adaptation and informs the conservation of threatened sky island species in the NCM.

## SUPPLEMENTARY DATA

Supplementary data to this article can be found online.

## Data Availability

The genome and whole-genome resequencing data used in this study can be retrieved from the following databases under accession code PRJCA042676 (https://ngdc.cncb.ac.cn/gwh/).
