# Supplementary material for: Survival at the edge: genomic vulnerability and genetic purging of a limestone cliff-endemic sky island shrub under climate change
Source: For Res (Fayettev). 2026 Apr 14;6:e013. doi: 10.48130/forres-0026-0010 (PMC13195435; doi:10.48130/forres-0026-0010)
Supplement: Supplementary file 1 — Supplementary data to this article can be found online. [file FR-2026-6-0010-S1.zip › 10.48130_forres-0026-0010-Suppl-FigureS12.pdf]

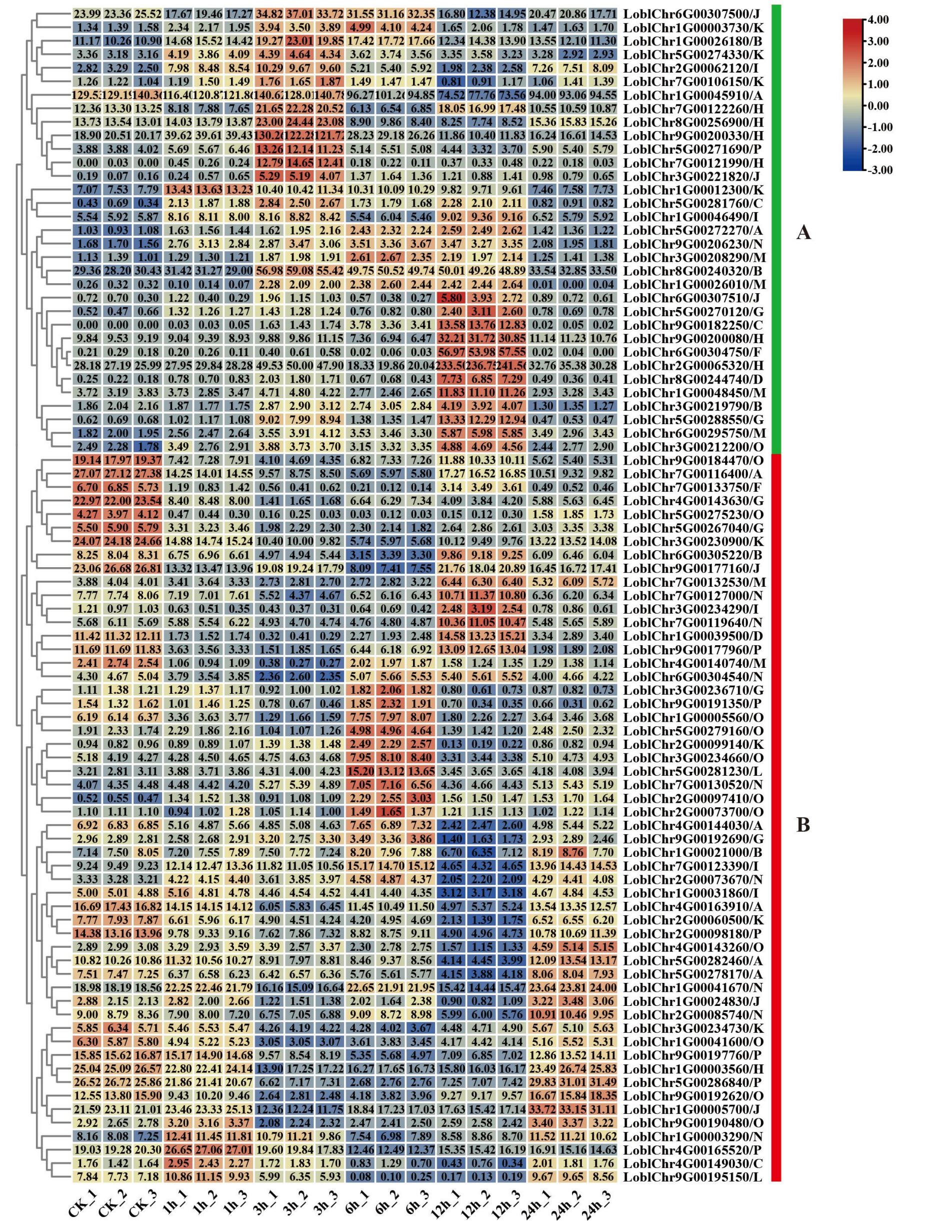

Figure S12. Heatmap showing expression profiles of *bHLH* genes in *L. oblata* under calcium stress treatment. The various colored bars on the left are used to distinguish five clusters. The scale indicates the relative signal intensity of FPKM values. The change from blue to red represents an increase in expression level.
