# Supplementary material for: Survival at the edge: genomic vulnerability and genetic purging of a limestone cliff-endemic sky island shrub under climate change
Source: For Res (Fayettev). 2026 Apr 14;6:e013. doi: 10.48130/forres-0026-0010 (PMC13195435; doi:10.48130/forres-0026-0010)
Supplement: Supplementary file 1 — Supplementary data to this article can be found online. [file FR-2026-6-0010-S1.zip › 10.48130_forres-0026-0010-Suppl-FigureS13.pdf]

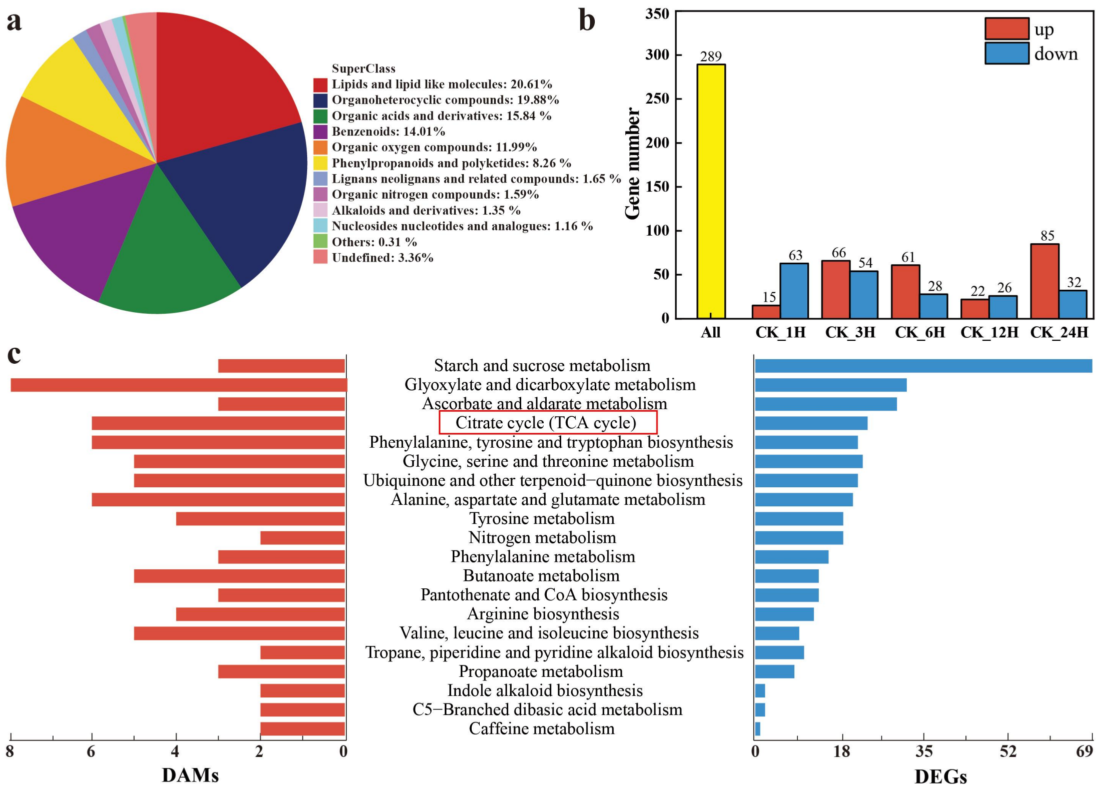

Figure S13. Metabolomics analysis of *Lonicera oblata* under calcium stress. (a) The proportion of metabolites identified by Superclass in each chemical classification (b) Numbers of upregulated and downregulated metabolites in five pairwise comparison groups. (c) The butterfly diagram of the KEGG pathways annotated by both DEGs and DAMs.
