# Supplementary material for: Survival at the edge: genomic vulnerability and genetic purging of a limestone cliff-endemic sky island shrub under climate change
Source: For Res (Fayettev). 2026 Apr 14;6:e013. doi: 10.48130/forres-0026-0010 (PMC13195435; doi:10.48130/forres-0026-0010)
Supplement: Supplementary file 1 — Supplementary data to this article can be found online. [file FR-2026-6-0010-S1.zip › 10.48130_forres-0026-0010-Suppl-FigureS14.pdf]

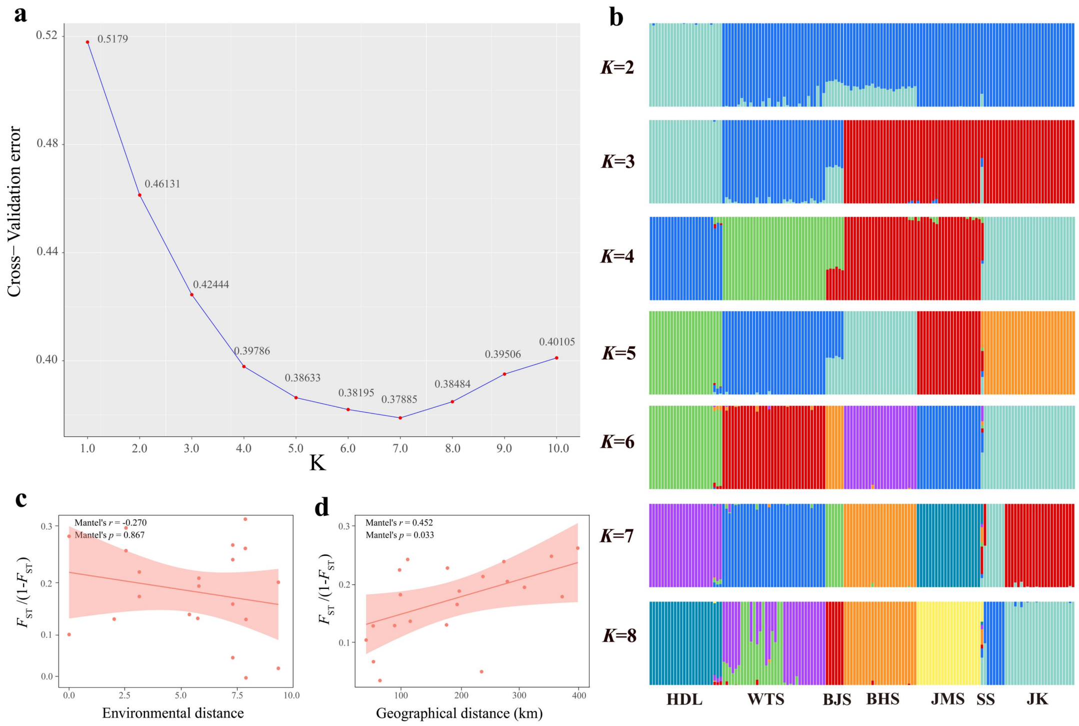

Figure S14. Results of the population structure and Mantel test in *Lonicera oblata*. (a) Distribution of cross-validation error according to the number of clusters (*K*) by Admixture. (b) Population structure of 140 *L. obalata* individuals at K = 2 to K=8. (c) Genetic vs. geographical distance correlation via Mantel test. (d) Genetic vs. environmental distance correlation via Mantel test.
