# Supplementary material for: Survival at the edge: genomic vulnerability and genetic purging of a limestone cliff-endemic sky island shrub under climate change
Source: For Res (Fayettev). 2026 Apr 14;6:e013. doi: 10.48130/forres-0026-0010 (PMC13195435; doi:10.48130/forres-0026-0010)
Supplement: Supplementary file 1 — Supplementary data to this article can be found online. [file FR-2026-6-0010-S1.zip › 10.48130_forres-0026-0010-Suppl-FigureS15.pdf]

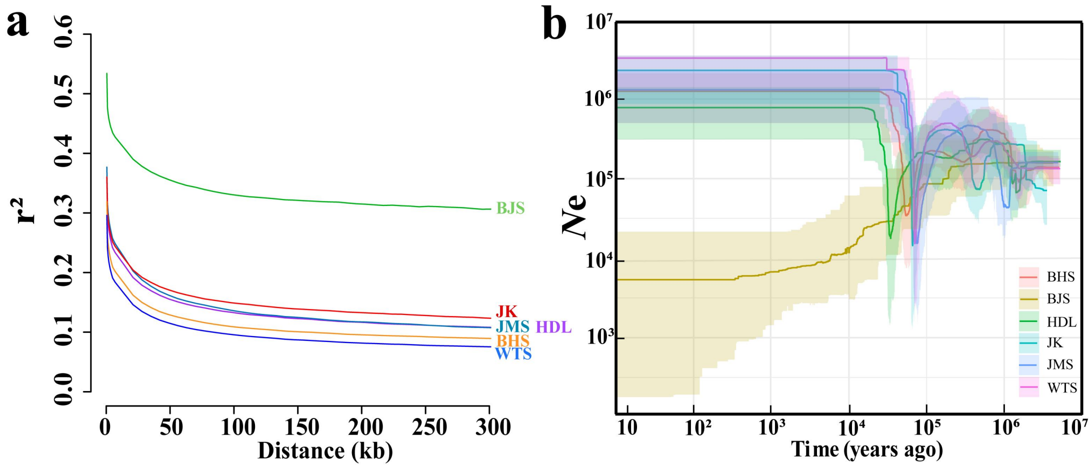


**Figure S15.** (a) Linkage disequilibrium decay of *Lonicera oblata*. (b) Demographic history of *L. oblata* inferred from Stairway Plot 2.
