# Supplementary material for: Survival at the edge: genomic vulnerability and genetic purging of a limestone cliff-endemic sky island shrub under climate change
Source: For Res (Fayettev). 2026 Apr 14;6:e013. doi: 10.48130/forres-0026-0010 (PMC13195435; doi:10.48130/forres-0026-0010)
Supplement: Supplementary file 1 — Supplementary data to this article can be found online. [file FR-2026-6-0010-S1.zip › 10.48130_forres-0026-0010-Suppl-FigureS16.pdf]

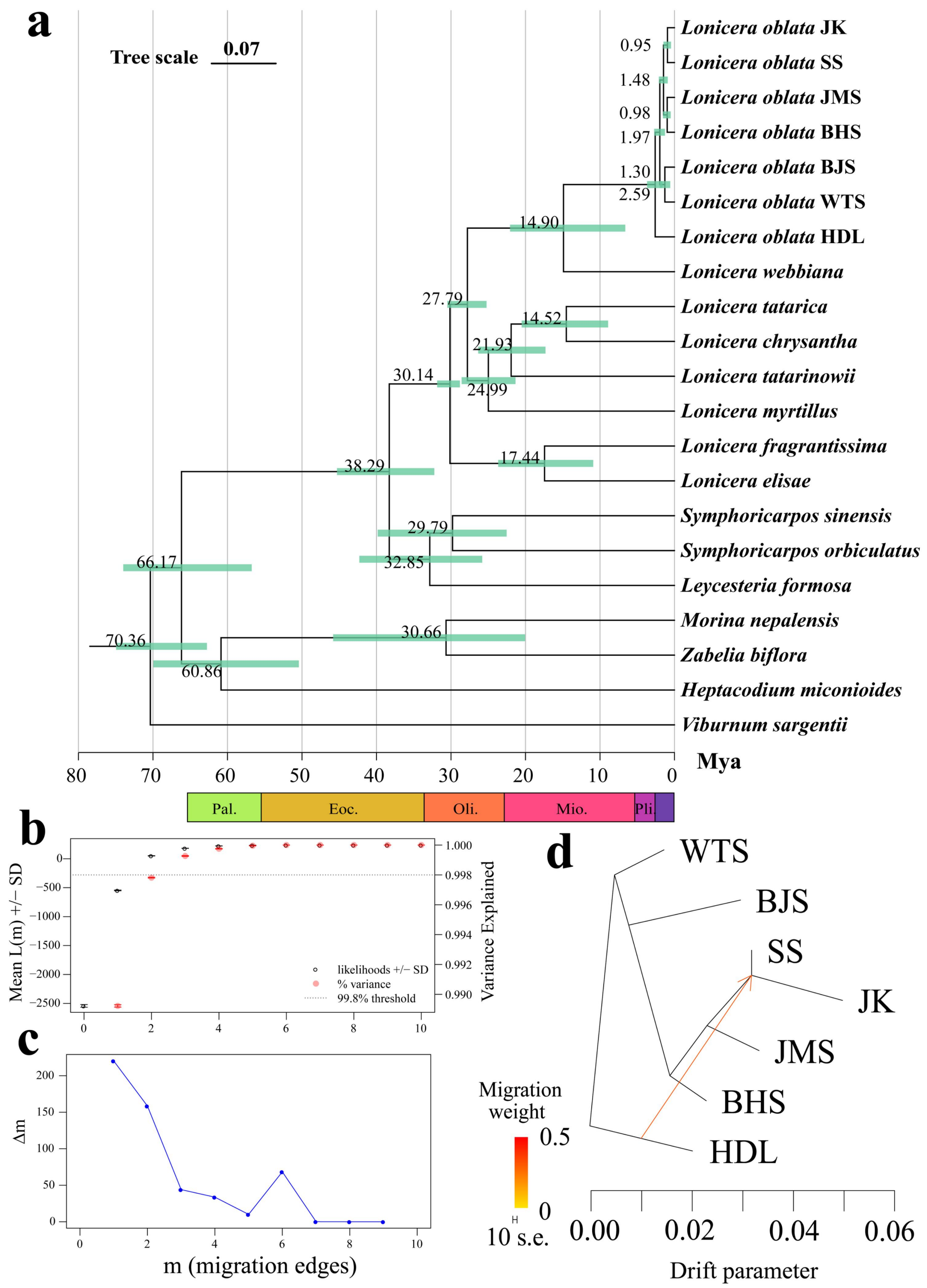

Figure S16. Dated phylogeny and gene flow of *Lonicera oblata*. (a) Dated phylogeny of *L. oblata* and Caprifoliaceae inferred from MCMCtree, with *Viburnum sargentii* serving as the outgroup. (b) Mean and standard deviation (SD) across 10 iterations for the composite likelihood function (m) (black circle) and proportion of variance explained (red dot). (c) Distribution of Δm inferred by R package OptM. (d) Gene flow scenarios inferred by Treemix at m = 1.
