# Supplementary material for: Survival at the edge: genomic vulnerability and genetic purging of a limestone cliff-endemic sky island shrub under climate change
Source: For Res (Fayettev). 2026 Apr 14;6:e013. doi: 10.48130/forres-0026-0010 (PMC13195435; doi:10.48130/forres-0026-0010)
Supplement: Supplementary file 1 — Supplementary data to this article can be found online. [file FR-2026-6-0010-S1.zip › 10.48130_forres-0026-0010-Suppl-FigureS18.pdf]

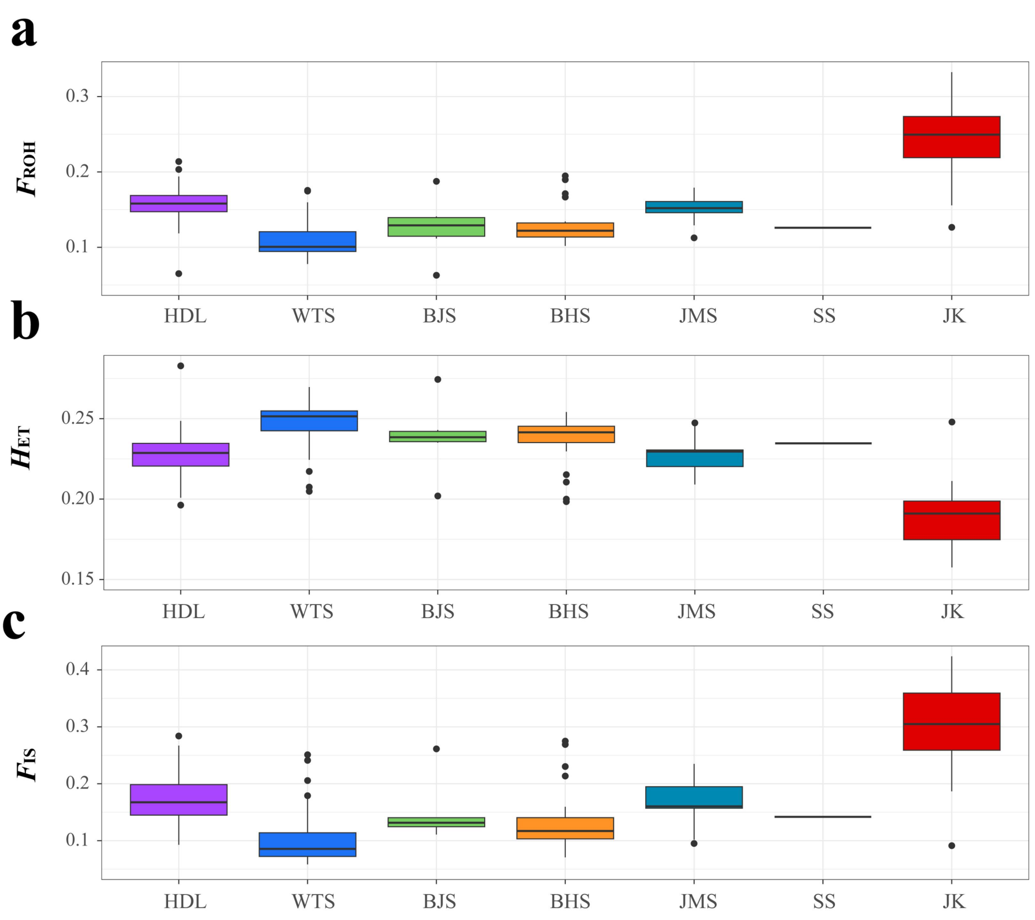

Figure S18. The fraction of the genome in *Lonicera oblata* populations. (a) the fraction of the genome in runs of homozygosity (F_{ROH}). (b) genomic heterozygosity (H_{ET}). (c) inbreeding coefficient (F_{IS}).
