# Supplementary material for: Survival at the edge: genomic vulnerability and genetic purging of a limestone cliff-endemic sky island shrub under climate change
Source: For Res (Fayettev). 2026 Apr 14;6:e013. doi: 10.48130/forres-0026-0010 (PMC13195435; doi:10.48130/forres-0026-0010)
Supplement: Supplementary file 1 — Supplementary data to this article can be found online. [file FR-2026-6-0010-S1.zip › 10.48130_forres-0026-0010-Suppl-FigureS2.pdf]

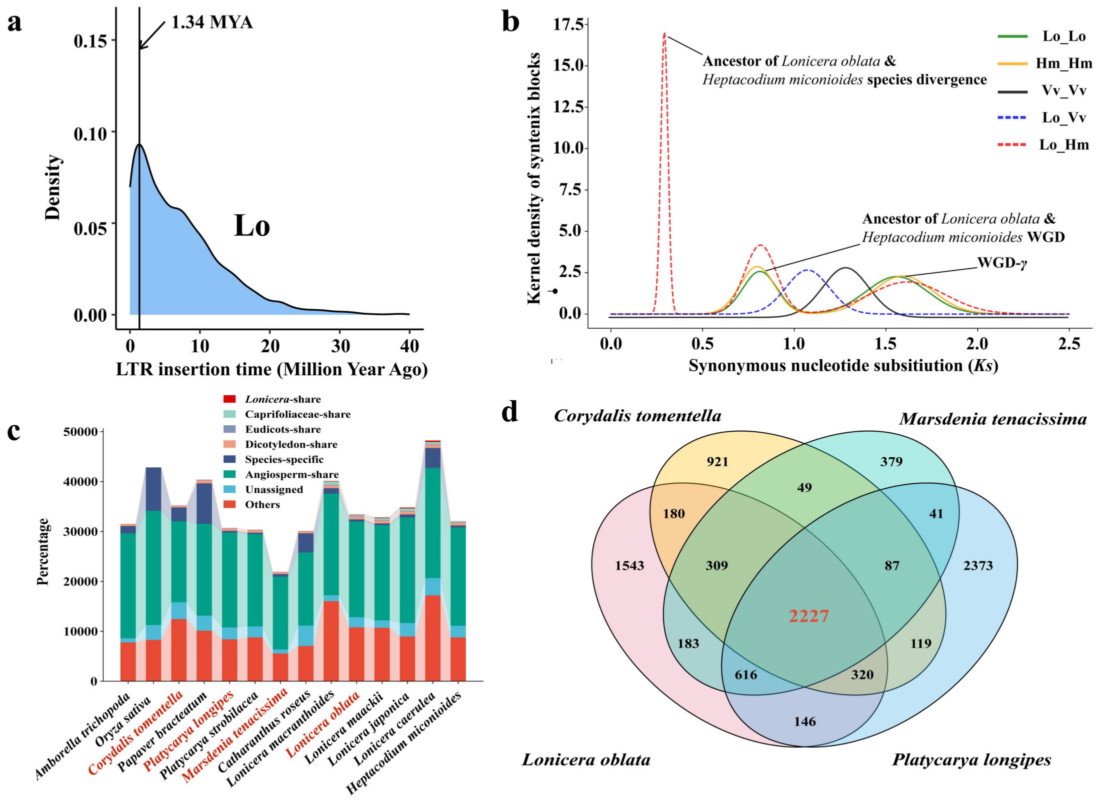

Figure S2. Comparative genomics reveals evolutionary adaptations in *Lonicera oblata*. (a) Analysis of LTR insertion times in the *L. oblata* genome. The abscissa represents the timing of LTR insertions. (b) Whole-genome duplication (WGD) events. The solid line shows the *Ks* distribution within the species, while the dashed line shows the *Ks* distribution among different species. *γ*-WGD events shared by all core eudicots are highlighted. (c) Cross-species orthogroup clustering across 14 angiosperm species. (d) Patterns of core gene conservation among limestone-endemic species. Red indicates the number of orthogroups that are commonly conserved among the four limestone-endemic plant species.
