# Supplementary material for: Survival at the edge: genomic vulnerability and genetic purging of a limestone cliff-endemic sky island shrub under climate change
Source: For Res (Fayettev). 2026 Apr 14;6:e013. doi: 10.48130/forres-0026-0010 (PMC13195435; doi:10.48130/forres-0026-0010)
Supplement: Supplementary file 1 — Supplementary data to this article can be found online. [file FR-2026-6-0010-S1.zip › 10.48130_forres-0026-0010-Suppl-FigureS20.pdf]

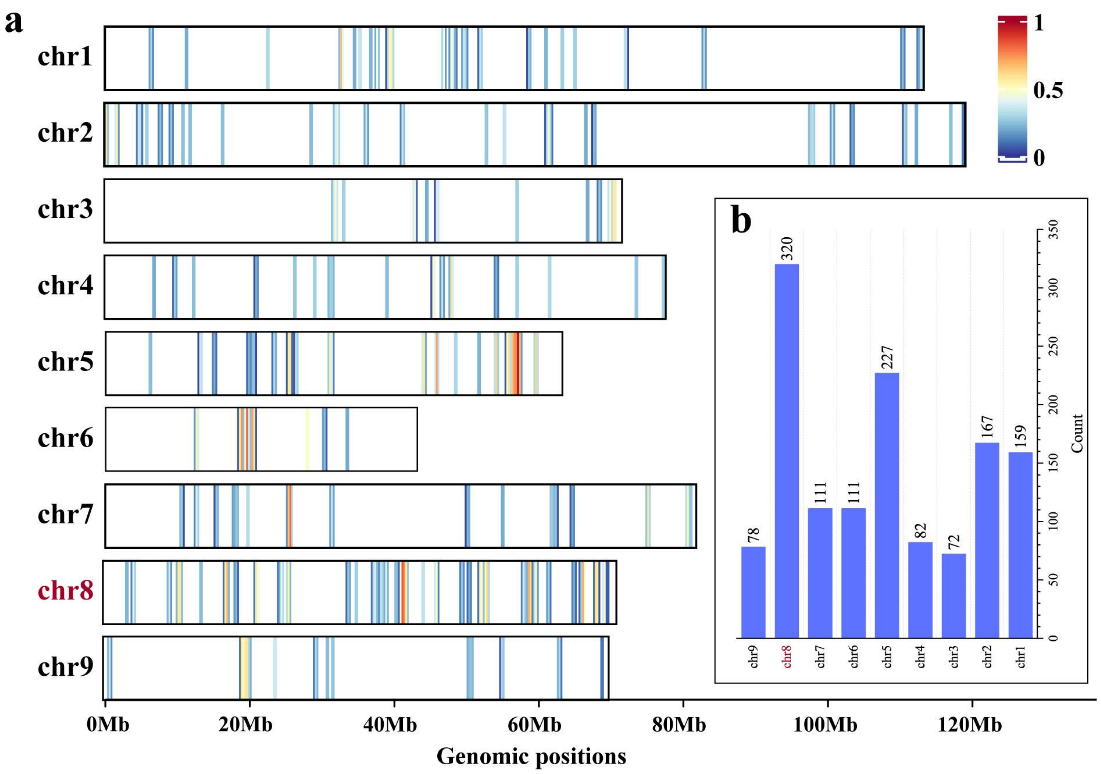

Figure S20. Density distribution and quantity statistics of the adaptive window on the chromosome in *Lonicera oblata*. (a) density distribution. (b) quantity statistics. Red indicates areas of higher density; blue indicates lower density. White regions show the background chromosomal distribution prior to adaptive window selection.
