# Supplementary material for: Survival at the edge: genomic vulnerability and genetic purging of a limestone cliff-endemic sky island shrub under climate change
Source: For Res (Fayettev). 2026 Apr 14;6:e013. doi: 10.48130/forres-0026-0010 (PMC13195435; doi:10.48130/forres-0026-0010)
Supplement: Supplementary file 1 — Supplementary data to this article can be found online. [file FR-2026-6-0010-S1.zip › 10.48130_forres-0026-0010-Suppl-FigureS21.pdf]

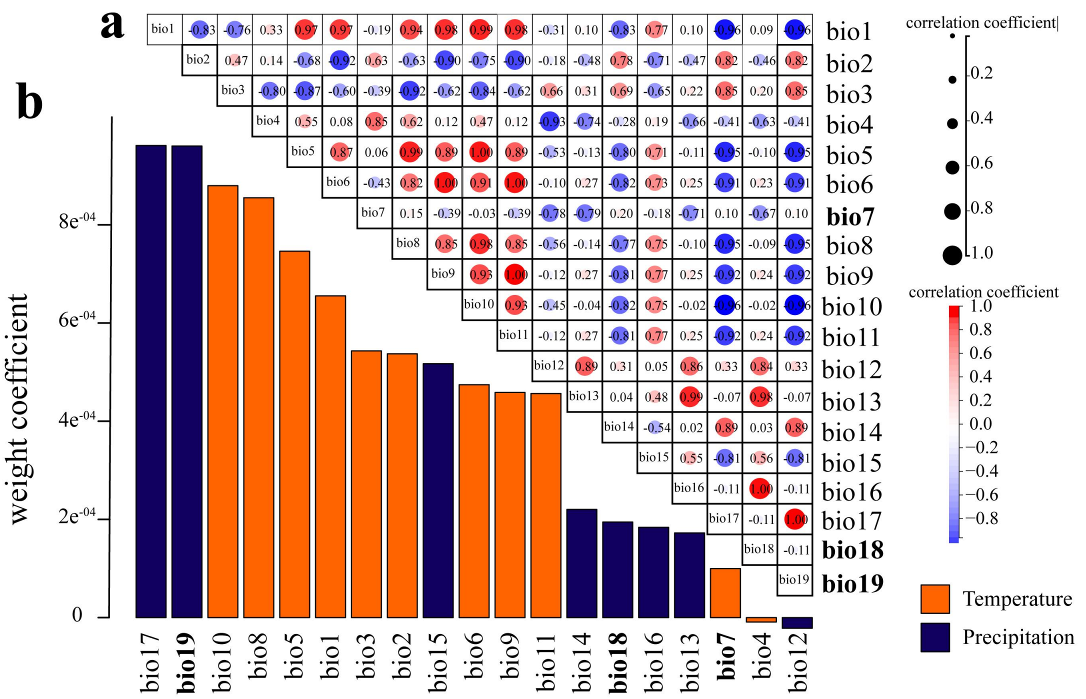

Figure S21. Screening of environmental indicators using gradient forest analysis and Pearson correlation. (a) Pearson correlation matrix of 19 bioclimatic variables at *Lonicera oblata* sampling sites. Red and blue indicate positive and negative correlations, respectively. Both bubble color intensity and size indicate absolute correlation strength (darker/larger = stronger correlation). (b) Importance ranking of the 19 bioclimatic variables based on gradient forest model. Variables are ordered left-to-right by decreasing importance weight. Temperature-associated variables are shown in dark blue; precipitation-associated variables in orange.
