# Supplementary material for: Survival at the edge: genomic vulnerability and genetic purging of a limestone cliff-endemic sky island shrub under climate change
Source: For Res (Fayettev). 2026 Apr 14;6:e013. doi: 10.48130/forres-0026-0010 (PMC13195435; doi:10.48130/forres-0026-0010)
Supplement: Supplementary file 1 — Supplementary data to this article can be found online. [file FR-2026-6-0010-S1.zip › 10.48130_forres-0026-0010-Suppl-FigureS23.pdf]

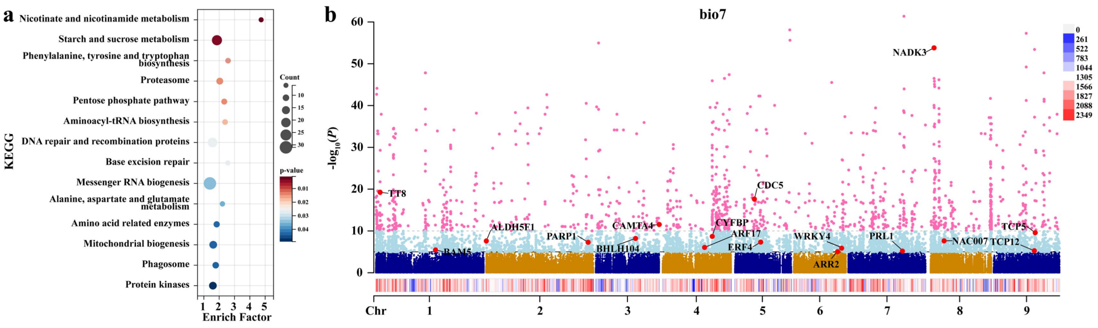

Figure S23. Genome-wide variation associated with environmental variable (bio7). (a) KEGG enrichment analysis for genes containing selective candidate SNPs. Horizontal axis shows enrichment fold change. Bubble size indicates gene count per pathway; color gradient represents *p*-value significance (blue to red means less to more significant). (b) Manhattan plot shows the genotype-environment association estimated with LFMM. Vertical axis shows the *p*-value of the relationship between SNPs and bio7, ranging from $p = 1$ to $p = 10^{-60}$. The light blue and pink dots indicate threshold values of $p = 10^{-5}$ and $p = 10^{-10}$, respectively. The colored strips with a red-blue gradient represent the density of SNPs on the chromosome.
