# Supplementary material for: Survival at the edge: genomic vulnerability and genetic purging of a limestone cliff-endemic sky island shrub under climate change
Source: For Res (Fayettev). 2026 Apr 14;6:e013. doi: 10.48130/forres-0026-0010 (PMC13195435; doi:10.48130/forres-0026-0010)
Supplement: Supplementary file 1 — Supplementary data to this article can be found online. [file FR-2026-6-0010-S1.zip › 10.48130_forres-0026-0010-Suppl-FigureS4.pdf]

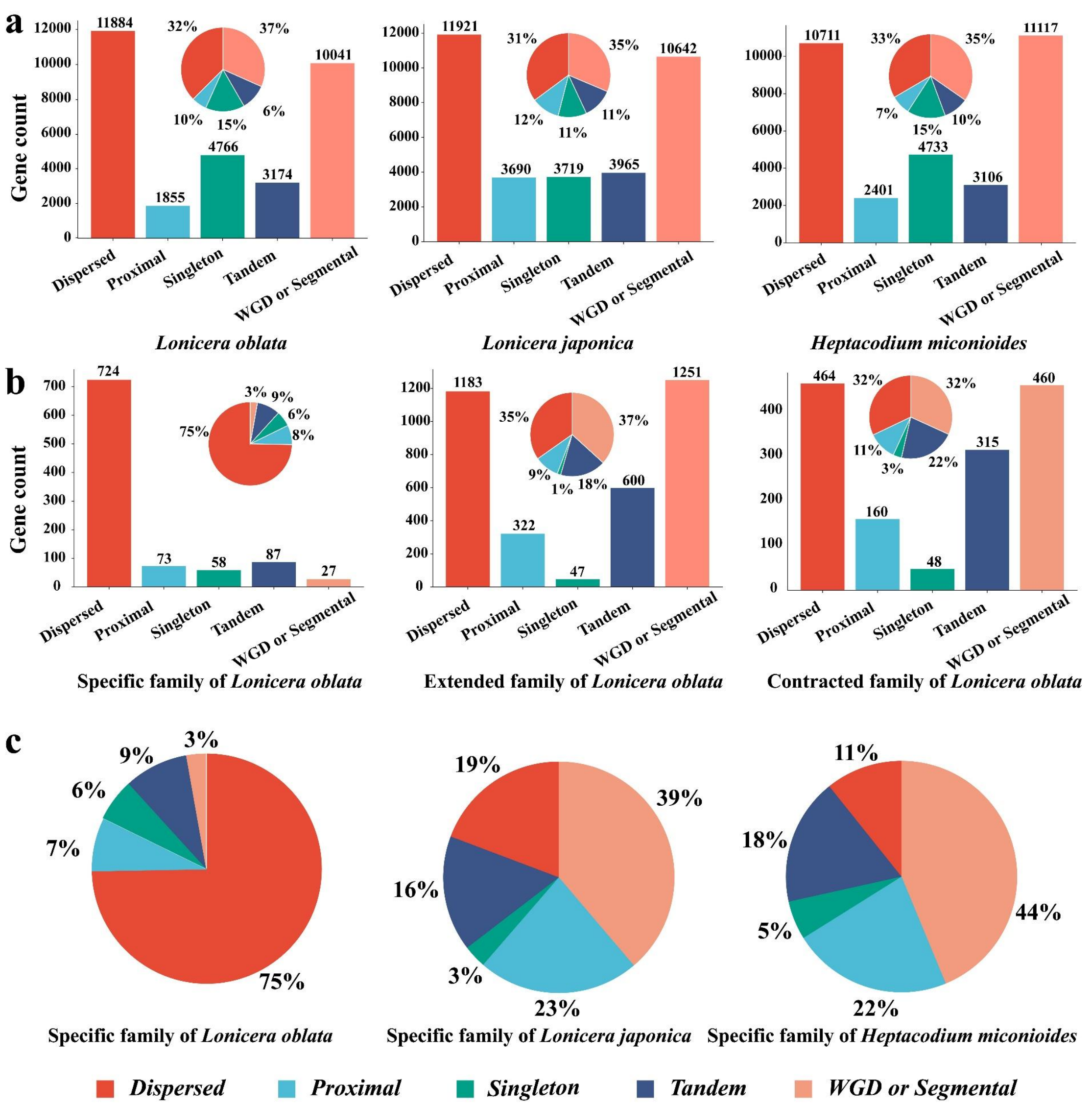


**Figure S4.** Analyses of duplication events. (a) Duplication events within Caprifoliaceae. (b) Duplication events across various families of *Lonicera oblata*. (c) Duplication events in specific families of *L. oblata*.
