# Supplementary material for: Survival at the edge: genomic vulnerability and genetic purging of a limestone cliff-endemic sky island shrub under climate change
Source: For Res (Fayettev). 2026 Apr 14;6:e013. doi: 10.48130/forres-0026-0010 (PMC13195435; doi:10.48130/forres-0026-0010)
Supplement: Supplementary file 1 — Supplementary data to this article can be found online. [file FR-2026-6-0010-S1.zip › 10.48130_forres-0026-0010-Suppl-FigureS6.pdf]

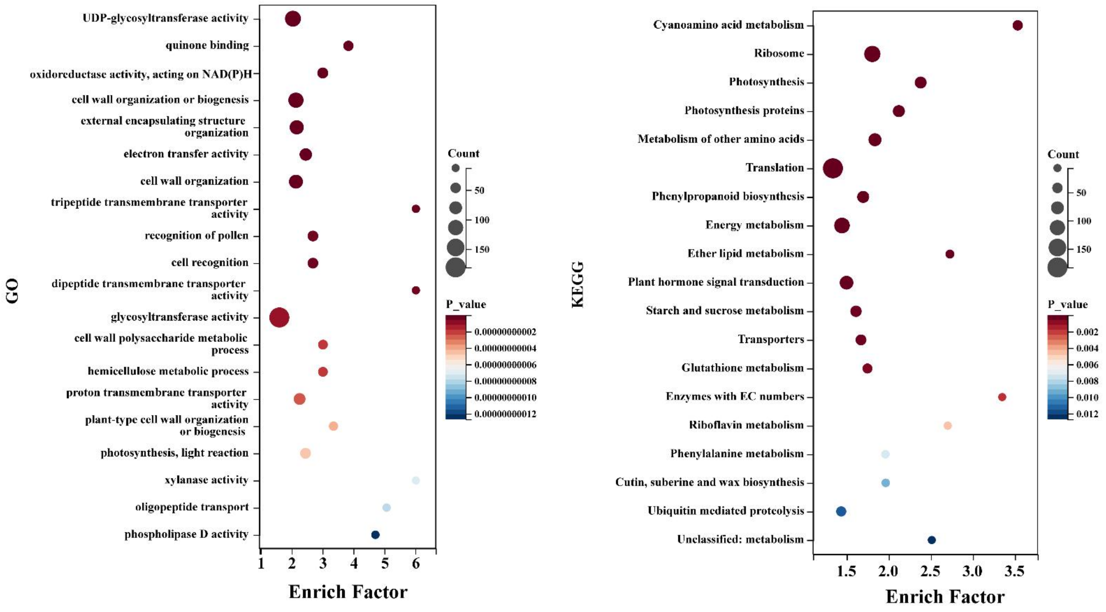

Figure S6. Functional enrichment analyses of the expanded orthogroups in *Lonicera oblata*. Left: GO enrichment results. Right: KEGG enrichment results. The horizontal axis represents the enrichment fold; the size of the bubbles indicates the number of genes in each pathway. The color gradient represents the *p*-value, transitioning from blue (less significant) to red (more significant).
