# Supplementary material for: Survival at the edge: genomic vulnerability and genetic purging of a limestone cliff-endemic sky island shrub under climate change
Source: For Res (Fayettev). 2026 Apr 14;6:e013. doi: 10.48130/forres-0026-0010 (PMC13195435; doi:10.48130/forres-0026-0010)
Supplement: Supplementary file 1 — Supplementary data to this article can be found online. [file FR-2026-6-0010-S1.zip › 10.48130_forres-0026-0010-Suppl-FigureS7.pdf]

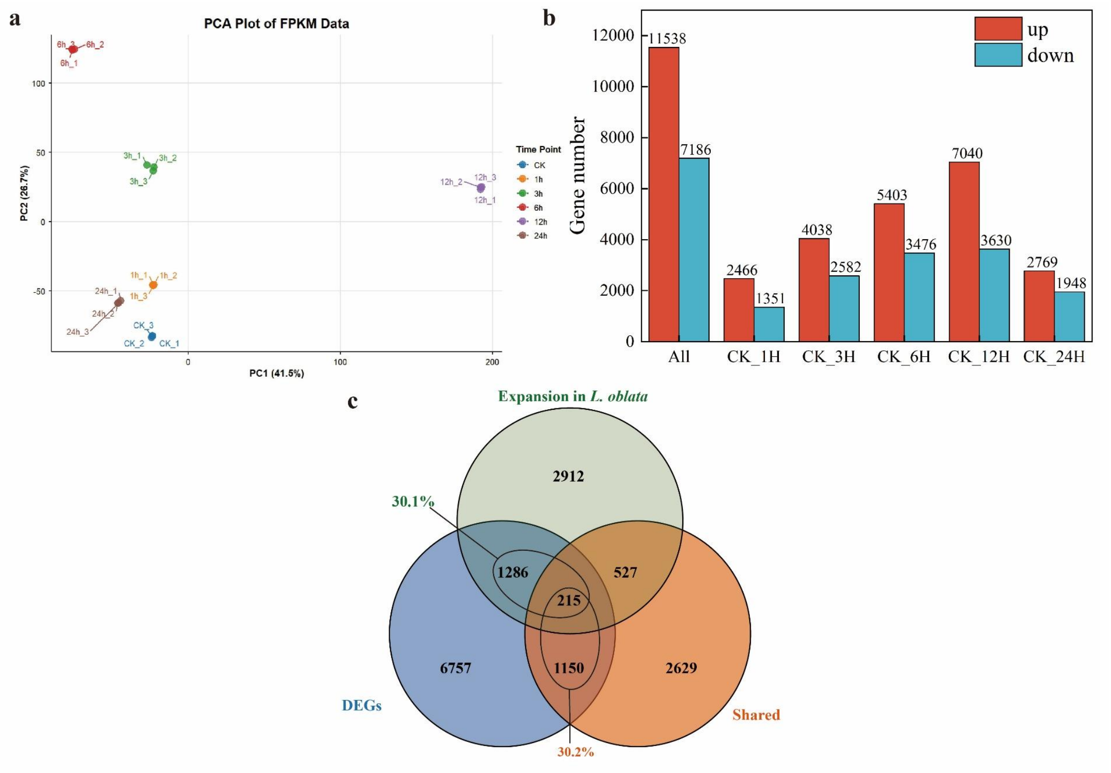

Figure S7. Transcriptome analysis of *Lonicera oblata* under calcium stress treatment. (a) PCA of transcriptome samples across six time points under calcium stress. Each time point is represented by a distinct color. (b) Numbers of upregulated and downregulated genes in five pairwise comparison groups. The total number of differentially expressed genes (DEGs) is shown. (c) Venn diagram showing the overlap between DEGs, expanded genes in *L. oblata*, and genes shared among four lithophytic species.
