# Supplementary figures and images for: Survival at the edge: genomic vulnerability and genetic purging of a limestone cliff-endemic sky island shrub under climate change
Source: For Res (Fayettev). 2026 Apr 14;6:e013. doi: 10.48130/forres-0026-0010 (PMC13195435; doi:10.48130/forres-0026-0010)

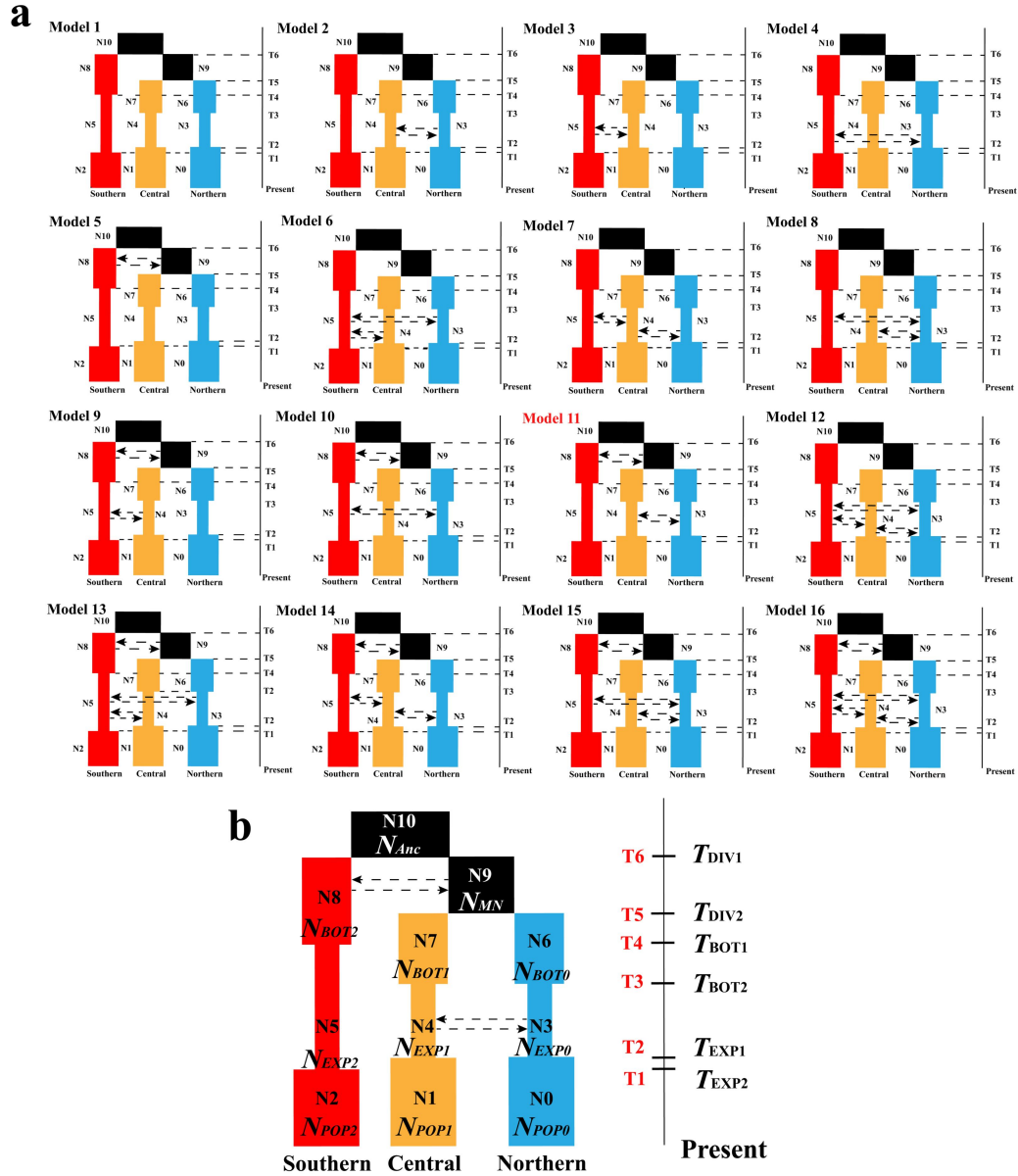

Supplement: Supplementary file 1 — Supplementary data to this article can be found online. [file FR-2026-6-0010-S1.zip › 10.48130_forres-0026-0010-Suppl-FigureS17.pdf]

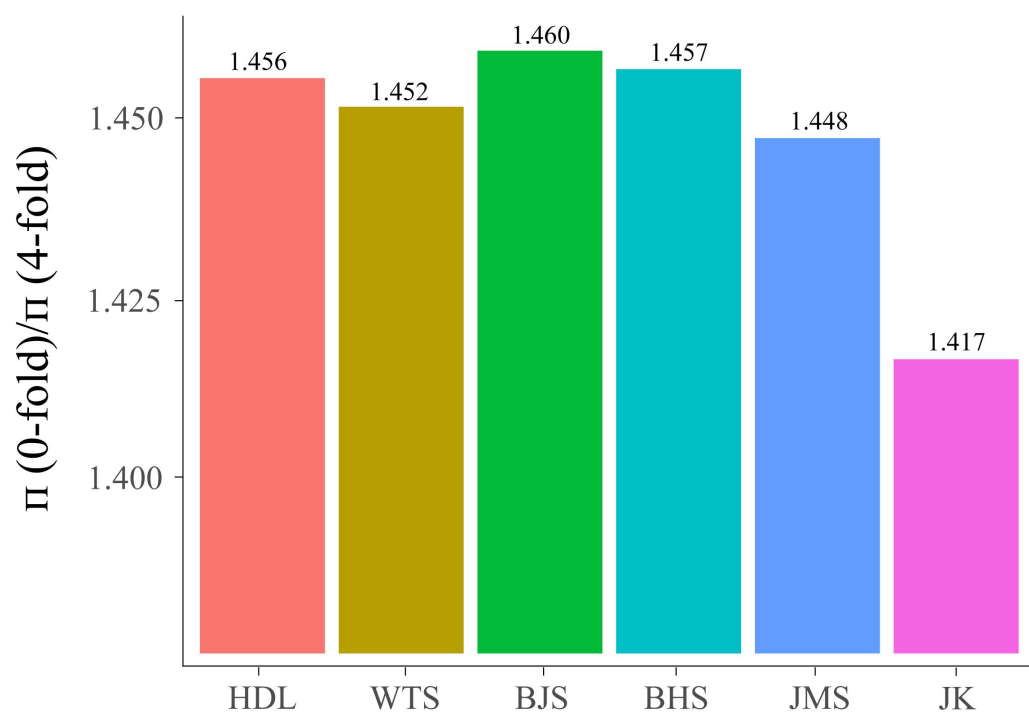

**Figure S19.** A comparison of  $\pi_0 / \pi_4$  ratios in *Lonicera oblata* populations.

Supplement: Supplementary file 1 — Supplementary data to this article can be found online. [file FR-2026-6-0010-S1.zip › 10.48130_forres-0026-0010-Suppl-FigureS19.pdf]

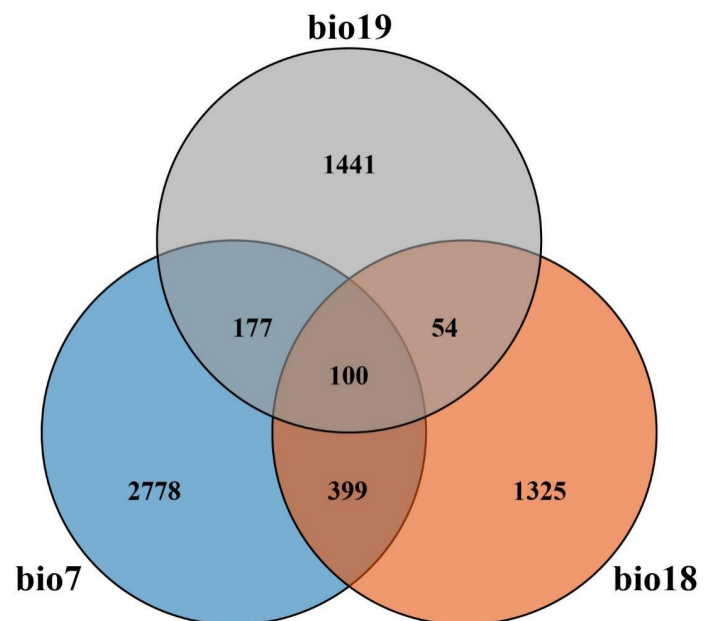

**Figure S22.** Venn diagram of shared association between three environmental variables and SNPs.

Supplement: Supplementary file 1 — Supplementary data to this article can be found online. [file FR-2026-6-0010-S1.zip › 10.48130_forres-0026-0010-Suppl-FigureS22.pdf]

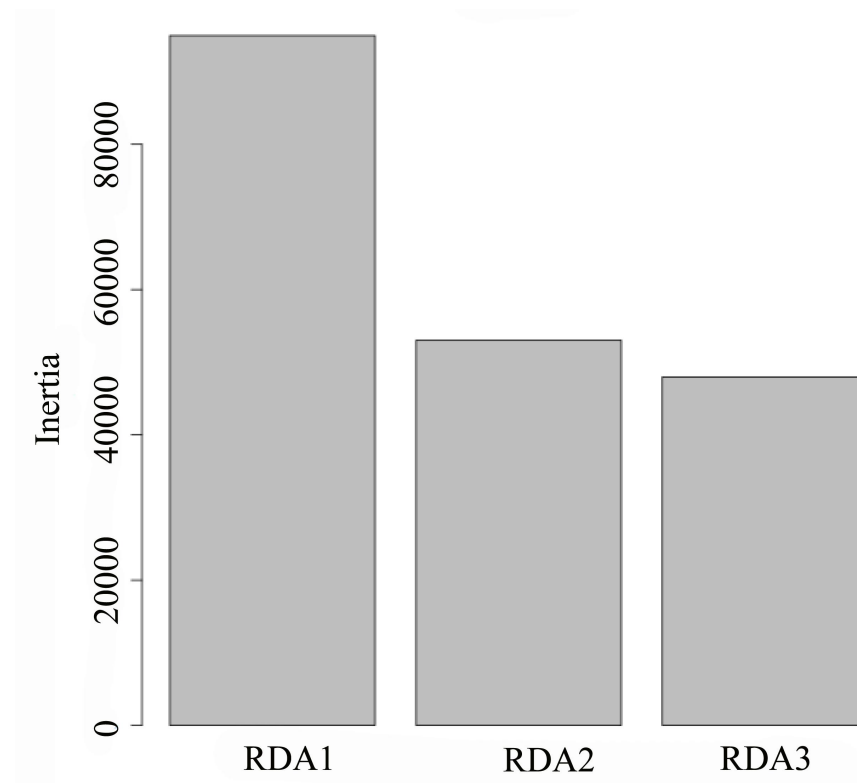

**Figure S25.** Variance explained by RDA axes.

Supplement: Supplementary file 1 — Supplementary data to this article can be found online. [file FR-2026-6-0010-S1.zip › 10.48130_forres-0026-0010-Suppl-FigureS25.pdf]

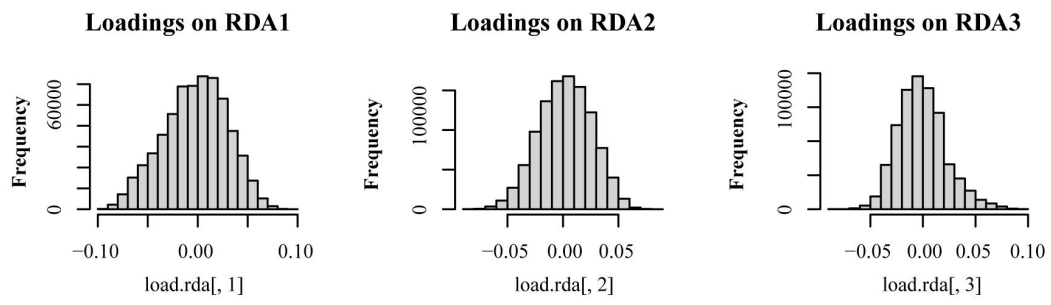

**Figure S26.** Variables loading distribution of the RDA1, RDA2, and RDA3 axes.

Supplement: Supplementary file 1 — Supplementary data to this article can be found online. [file FR-2026-6-0010-S1.zip › 10.48130_forres-0026-0010-Suppl-FigureS26.pdf]

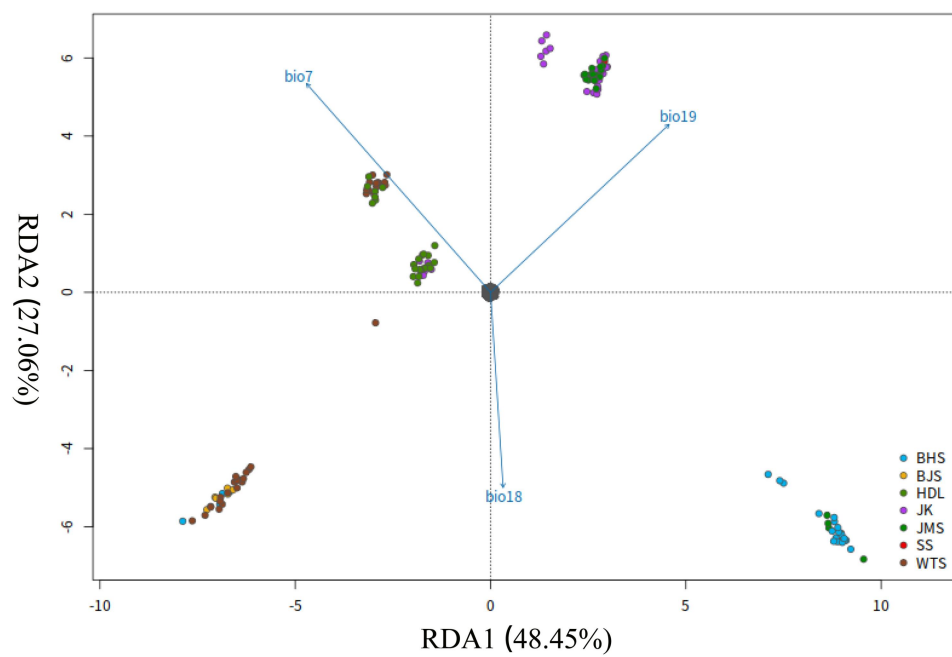

**Figure S27.** PCA plot based on RDA axes 1 and 2.

Supplement: Supplementary file 1 — Supplementary data to this article can be found online. [file FR-2026-6-0010-S1.zip › 10.48130_forres-0026-0010-Suppl-FigureS27.pdf]
